# At‐RS31 orchestrates hierarchical cross‐regulation of splicing factors and integrates alternative splicing with TOR‐ABA pathways

**DOI:** 10.1111/nph.70221

**Published:** 2025-05-26

**Authors:** Tino Köster, Peter Venhuizen, Martin Lewinski, Ezequiel Petrillo, Yamile Marquez, Armin Fuchs, Debashish Ray, Barbara A. Nimeth, Stefan Riegler, Sophie Franzmeier, Florencia S. Rodríguez, Federico E. Aballay, Rocío S. Tognacca, Hong Zheng, Timothy Hughes, Quaid Morris, Andrea Barta, Dorothee Staiger, Maria Kalyna

**Affiliations:** ^1^ RNA Biology and Molecular Physiology, Faculty of Biology Bielefeld University Bielefeld 33615 Germany; ^2^ Institute of Molecular Plant Biology, Department of Biotechnology and Food Science BOKU University Vienna 1190 Austria; ^3^ Instituto de Fisiología Biología Molecular y Neurociencias (IFIBYNE) Buenos Aires 1428 Argentina; ^4^ Max Perutz Labs Medical University Vienna, Vienna BioCenter (VBC) Vienna 1030 Austria; ^5^ Donnelly Centre University of Toronto Toronto ON M5S 3E1 Canada; ^6^ Department of Molecular Genetics University of Toronto Toronto ON M5S 3E1 Canada; ^7^ Sloan Kettering Institute Memorial Sloan Kettering Cancer Center New York NY 10065 USA; ^8^ Graduate Program in Computational Biology and Medicine Weill‐Cornell Graduate School New York NY 10065 USA

**Keywords:** ABA, alternative splicing, *Arabidopsis thaliana*, binding site, iCLIP, RNAcompete, SR proteins, TOR kinase

## Abstract

- Alternative splicing is essential for plants, enabling a single gene to produce multiple transcript variants to boost functional diversity and fine‐tune responses to environmental and developmental cues. *Arabidopsis thaliana* At‐RS31, a plant‐specific splicing factor in the Serine/Arginine‐rich (SR) protein family, responds to light and the Target of Rapamycin (TOR) signalling pathway, yet its downstream targets and regulatory impact remain unknown.
- To identify At‐RS31 targets, we applied individual‐nucleotide resolution crosslinking and immunoprecipitation (iCLIP) and RNAcompete assays. Transcriptomic analyses of At‐RS31 mutant and overexpressing plants further revealed its effects on alternative splicing.
- iCLIP identified 4034 At‐RS31 binding sites across 1421 genes, enriched in CU‐rich and CAGA RNA motifs. Comparative iCLIP and RNAcompete data indicate that the arginine/serine (RS) domain of At‐RS31 may influence its binding specificity *in planta*, underscoring the value of combining *in vivo* and *in vitro* approaches. Transcriptomic analysis showed that At‐RS31 modulates diverse splicing events, particularly intron retention and exitron splicing, and influences other splicing modulators, acting as a hierarchical regulator.
- By regulating stress response genes and genes in both TOR and abscisic acid signalling pathways, At‐RS31 may help integrate these signals, balancing plant growth with environmental adaptability through alternative splicing.

## Introduction

Gene expression in eukaryotes involves multiple regulatory layers. Following transcription, nascent RNAs undergo processing steps, including capping, splicing, polyadenylation, and chemical modification, to produce mature mRNAs (Yang *et al*., 2021). Splicing removes introns from pre‐mRNA and joins exons to generate the mature transcripts (Gilbert, 1978). Although splicing is a highly regulated process ensuring specificity, it also shows remarkable plasticity. The spliceosome, the cellular machinery responsible for splicing, can recognize alternative splice sites, enabling a single gene to produce multiple transcript variants via alternative splicing.

In plants, 40–70% of intron‐containing genes undergo alternative splicing, underscoring its fundamental role in regulating gene expression during development and environmental responses (Filichkin *et al*., 2010; Lu *et al*., 2010; Marquez *et al*., 2012; Chamala *et al*., 2015). Alternative splicing not only produces diverse transcripts leading to different proteins but also generates noncoding isoforms, which may be rapidly degraded or remain stable, thus fine‐tuning the total protein levels produced by a gene (Kalyna *et al*., 2012; Petrillo, 2023). Different types of alternative splicing events, such as exon skipping (ES), intron retention, and usage of alternative 5′ and 3′ splice sites, generate transcript diversity. While ES is common in animals, intron retention is most frequent in plants. Retained intron (RI) transcripts often remain in the nucleus, regulating protein levels during stress or developmental transitions (Kalyna *et al*., 2012; Marquez *et al*., 2012; Yap *et al*., 2012; Boothby *et al*., 2013; Leviatan *et al*., 2013; Braunschweig *et al*., 2014; Gohring *et al*., 2014; Boutz *et al*., 2015). Furthermore, exitrons (EIs), alternatively spliced internal regions within protein‐coding exons, add another layer of complexity to the alternative splicing landscape (Marquez *et al*., 2015; Staiger & Simpson, 2015).

The spliceosome ensures the accurate recognition of different pre‐mRNA regions and intron removal, aided by numerous proteins. Among these proteins, two key groups stand out: serine/arginine‐rich (SR) proteins and heterogeneous nuclear ribonucleoproteins (hnRNPs; Wachter *et al*., 2012). Serine/arginine‐rich proteins interact with the pre‐mRNA and spliceosomal components, guiding spliceosome assembly at specific splice sites (Shepard & Hertel, 2009). They contain one or two *N*‐terminal RNA recognition motifs (RRMs), the most prevalent RNA‐binding domain, and a C‐terminal arginine/serine (RS) region enriched in arginine/serine dipeptides, which engages primarily in protein–protein interactions but also contributes to RNA recognition. By interacting with the spliceosomal machinery, SR proteins modulate splice site selection, contributing to mRNA isoform diversity. Beyond splicing, SR proteins influence transcription (Lin *et al*., 2008; Ji *et al*., 2013), polyadenylation (Schwich *et al*., 2021), mRNA export (Müller‐McNicoll *et al*., 2016; Botti *et al*., 2017), translation (Sanford *et al*., 2004) among other processes. However, most knowledge about SR protein functions comes from animal studies.

In plants, the SR protein family has expanded remarkably, with *Arabidopsis thaliana* possessing 18 SR proteins classified into six subfamilies. Ten are plant‐specific, divided into RS, RS2Z, and SCL subfamilies based on their domain organization. The remaining eight are similar to mammalian SR proteins SF2/ASF/SRSF1, 9G8/SRSF7, and SC35/SRSF2 and belong to SR, RSZ, and SC subfamilies, respectively (Kalyna & Barta, 2004; Barta *et al*., 2008, 2010; Duque, 2011; Richardson *et al*., 2011). Arabidopsis also has two SR‐like proteins: SR45 and SR45a. These proteins participate in constitutive and alternative splicing and play roles in mRNA export, stability, translation, transcriptional elongation, and cell cycle regulation (Jin, 2022). Several SR proteins contribute to plant development and abiotic stress responses (Jin, 2022), but their *in vivo* targets and regulatory networks remain less characterized (Mateos & Staiger, 2023). So far, RNA immunoprecipitation (RIP) followed by RNA sequencing (RNA‐seq) identified over 4000 RNAs associated with SR45 in Arabidopsis seedlings (Xing *et al*., 2015) and 1812 in inflorescences (Zhang *et al*., 2017). Recently, tomato RS2Z35 and RS2Z36 were shown to bind to transcripts of over 5000 genes, including the heat shock transcription factor (TF) and many transcripts that undergo heat shock‐sensitive alternative splicing, preferentially binding purine‐rich RNA motifs (Rosenkranz *et al*., 2024).

At‐RS31 (AT3G61860), a plant‐specific SR protein in the RS subfamily, may regulate unique plant functions, although its exact roles are unclear. It has two *N*‐terminal RRMs and the RS region, specific to this subfamily (Supporting Information Fig. S1a; Lopato *et al*., 1996). At‐RS31 interacts with SR and SR‐like proteins and spliceosome components, suggesting a role in pre‐mRNA splicing (Lopato *et al*., 2002; Lorkovic *et al*., 2005; Altmann *et al*., 2020). Its ability to stimulate splicing in SR protein‐deficient HeLa cell S100 extracts further supports this role (Lopato *et al*., 1996).

*At‐RS31* undergoes alternative splicing, producing four transcript isoforms: mRNA1‐4 (Lopato *et al*., 1996; Fig. S1b,c). The shortest isoform, mRNA1, arises from excision of the entire intron 2 and encodes the SR protein. This whole intron is retained in mRNA4. mRNA3 uses a proximal 3′ splice site in intron 2, while mRNA2 arises either from the removal of a small intron in mRNA3 or from the inclusion of a cassette exon compared with mRNA1. mRNA2–4 contain premature termination codons (PTCs). Only mRNA2 is sensitive to nonsense‐mediated mRNA decay (NMD), likely due to nuclear retention of mRNA3 and mRNA4 (Kalyna *et al*., 2012; Petrillo *et al*., 2014).

The ratio of *At‐RS31* isoforms varies with tissue type, developmental stage, and environmental stimuli, such as bacterial flagellin, cold, or red light (Lopato *et al*., 1996; Palusa *et al*., 2007; Tognacca *et al*., 2019; Bazin *et al*., 2020). The proportion of mRNA1 fluctuates in response to light, increasing in light and decreasing in darkness, a response mediated by chloroplast retrograde signalling, affecting even nonphotosynthetic root cells lacking chloroplasts; sugars, mitochondrial function, and the Target of Rapamycin (TOR) pathway are key to this effect (Petrillo *et al*., 2014; Riegler *et al*., 2021). The conserved alternative splicing pattern of *At‐RS31* across diverse plant species, from green algae to flowering plants, underscores its biological significance (Iida & Go, 2006; Kalyna *et al*., 2006).

Despite the identification of many factors regulating *At‐RS31* alternative splicing, hinting at its potential roles in several plant biological processes, its downstream targets remain unknown. Since SR proteins influence alternative splicing in a concentration‐dependent manner (Mayeda *et al*., 1992) and given the dynamic modulation of *At‐RS31* in response to various environmental and developmental signals, we hypothesize that its expression levels significantly impact the transcriptome. To identify direct targets of At‐RS31, we performed *in vivo* individual‐nucleotide resolution crosslinking and immunoprecipitation (iCLIP; Konig *et al*., 2010; Hafner *et al*., 2021) followed by *in vitro* RNAcompete validation (Ray *et al*., 2013, 2017). Additionally, we performed transcriptome profiling in *rs31‐1* mutant plants and plants constitutively overexpressing At‐RS31 (Petrillo *et al*., 2014). By identifying At‐RS31 direct targets through iCLIP and transcripts that are differentially alternatively spliced in response to altered At‐RS31 levels, we gain insights into the downstream processes controlled by At‐RS31.

## Materials and Methods

### Generation of green fluorescent protein‐tagged *At‐RS31* genomic construct and transgenic plants for iCLIP

To generate *RS31::RS31‐GFP* construct for iCLIP (Fig. S2a), the *At‐RS31* genomic region including its own promoter, untranslated regions (UTRs), and introns was amplified from *Arabidopsis thaliana* (L.) Heynh. Columbia‐0 (Col‐0) DNA by polymerase chain reaction (PCR). The construct was cloned into pGreenII0029. C‐terminal fusion to green fluorescent protein (GFP) was achieved by mutating the *At‐RS31* reference stop codon via site‐directed PCR mutagenesis. The resulting construct was used to generate transgenic *A. thaliana* plants via floral dipping (Clough & Bent, 1998). The *RS31::RS31‐GFP* construct was introduced into the *rs31‐1* mutant line (see Fig. S1b). Wild‐type (WT) plants were transformed with the *35S::GFP* construct, selected on 50 μg l^−1^ kanamycin, and by genotyping. Transgenic plants containing *RS31::RS31‐GFP* were selected via fluorescence stereomicroscopy and genotyping. Genomic DNA was isolated from a single leaf as described by Edwards *et al*. (1991), and genotyping was performed using primers listed in Table S1.

Transgenic plants were assessed for the expression of the At‐RS31‐GFP fusion protein. Plant tissues were ground in liquid nitrogen and resuspended in protein extraction buffer (PEB) 400 (50 mM 4‐(2‐hydroxyethyl)‐1‐piperazineethanesulfonic acid–potassium hydroxide pH 7.9, 400 mM potassium chloride (KCl), 2.5 mM magnesium chloride, 1 mM ethylenediaminetetraacetic acid pH 8.0, 1 mM dithiothreitol, 0.1% Tween‐20, protease, and phosphatase inhibitor). The crude extract was incubated on ice for 15 min before sonication, followed by three rounds of centrifugation. The final supernatant was diluted with PEB KCl‐free buffer to adjust the KCl concentration to 200 mM. Western blotting was performed according to standard procedures. Proteins were separated by 10–16% sodium dodecyl sulphate–polyacrylamide gel electrophoresis (SDS‐PAGE). Immunoblotting (Fig. S2b) was performed using rabbit monoclonal anti‐GFP (D5.1) antibody (Cell Signaling Technology Europe B.V., Leiden, The Netherlands) at a 1:1000 dilution.

### Confocal laser scanning microscopy

*Arabidopsis thaliana* mesophyll protoplasts were isolated as described in Wu *et al*. (2009) from fully expanded leaves from 3‐wk‐old plants of WT, 35S::GFP, RS31::RS31‐GFP, and 35S::RS31‐GFP lines (grown under a 16 h : 8 h, light : dark cycle at 22°C in soil). Protoplasts were resuspended in W5 medium and incubated at 22°C for 16 h before the analysis of transgene expression. Protoplasts were analysed using a Zeiss LSM700 laser scanning confocal microscope in the dual‐track channel mode. Green fluorescent protein and chloroplast autofluorescence were excited with a 488‐nm laser line, and emissions were recorded at 505–530 and 650–710 nm, respectively. Images were unmixed *in silico* using previously recorded, background‐free spectra of chloroplast autofluorescence and of GFP.

### Growth of plant material for iCLIP

Seeds of RS31::RS31‐GFP and 35S::GFP plants were sown on agar plates containing ½‐strength Murashige & Skoog medium (½MS): 2.2 g l^−1^ MS (M0222.0050; Duchefa, Haarlem, The Netherlands), 0.5 g l^−1^ 2‐morpholinoethanesulfonic acid (MES), 1% sucrose, and 1% agar. The plates were placed vertically in growth cabinets and incubated under a 16 h : 8 h, light : dark cycle at 22°C and 60% humidity for 14 d.

### Individual‐nucleotide resolution crosslinking and immunoprecipitation

RS31::RS31‐GFP and 35S::GFP seedlings were subjected to irradiation with 254 nm ultraviolet (UV)‐light at a dose of 2000 mJ cm^−2^ (UVP CL‐1000 UV crosslinker) on ice 4 h after lights on. iCLIP was performed essentially as described (Meyer *et al*., 2017; Köster & Staiger, 2021). Adapter sequences are provided in Table S1.

### Bioinformatics evaluation of iCLIP reads

The quality of sequenced reads was examined using fastqc v.0.11.5 (http://www.bioinformatics.bbsrc.ac.uk/projects/fastqc). Adapters at the 3′ end were trimmed using cutadapt v.1.16 (Martin, 2011). The samples were then demultiplexed with the flexbar toolkit v.3.4.0 by applying the additional ‐bk parameter to conserve the barcode information for further steps (Roehr *et al*., 2017). Reads shorter than 24 nucleotides (nt) were discarded. Barcodes were manually trimmed and saved to the *read_id* field. The processed reads were mapped to the TAIR10 genome with star v.2.6.0a, allowing a maximum of two mismatches and soft clipping only at the 3′ end (Dobin *et al*., 2013). The removal of PCR duplicates was performed by grouping the reads by their mapping start position. Reads with the identical start position and random barcode were removed from the samples (python3 and pybedtools; Dale *et al*., 2011). The peak calling of uniquely mapped reads was performed using pureclip v.1.0.4 (Krakau *et al*., 2017). The PureCLIP parameters were set to the second profile option (−bc 1) allowing broader regions with less read starts to be called. To remove redundancy after the peak calling, peaks located at immediately adjacent nucleotides were grouped together and only the peak with the highest PureCLIP score was kept. The called peak position was extended by 4 nt upstream and downstream to define a binding site of 9 nt. The centre position of the binding site marks the binding site peak. The extension of the binding site peak positions was computed using bedtools v.2.27.1 (Quinlan & Hall, 2010).

### Motif discovery

The sequence at each binding site (length of 9 nt) was extracted by applying the getfasta program from bedtools (Quinlan & Hall, 2010) in a strand‐specific mode (−s) using TAIR10 as the reference genome. A *de novo* motif discovery was then applied using streme v.5.3.3 (Bailey, 2021) to identify significantly enriched motifs with a length between 3 and 8 nt in the binding site sequences. Additionally, *k*‐mer distributions with the identical set of binding site sequences were computed using *k* = 6 (hexamers) similar to the length of significant motifs identified by streme. To account for local sequence bias, the hexamer counts were normalized by sampling sequences randomly 1000 times from the identical transcripts targeted by the RS31‐GFP protein. The randomized set was used to calculate a *z*‐score for each hexamer by subtracting the mean of random occurrences from the actual occurrence and dividing it by the SD of the random occurrences.

### Binding site distribution in protein‐coding genes

Each binding site was assigned to the overlapping transcript and gene feature, namely 5′ UTR, coding sequence (CDS), intron, or 3′ UTR according to the Araport11 annotation. Only the representative models from TAIR of a given gene were considered to avoid ambiguity in the assignment. The distribution of binding sites among gene features from RS31::RS31‐GFP and 35S::GFP samples was compared using the percentage of mapped binding sites.

To determine the location of At‐RS31 binding sites relative to the 5′ splice site, the peak positions of all binding sites were compared against 5′ splice sites from protein‐coding genes annotated in Araport11 using a custom R Script and the tidyverse package (https://www.tidyverse.org/). Only exons with a length of at least 50 nt were considered. Sequences around At‐RS31 binding sites 20 to 35 nt upstream of 5′ splice sites were extracted using getfasta from bedtools (Quinlan & Hall, 2010) and aligned using the ClustalO Web Service from jalview v.2.11.1.7 (Waterhouse *et al*., 2009). Based on the resulting alignments, a sequence logo was created using weblogo3 (Crooks *et al*., 2004).

The positional relationship of At‐RS31 binding sites to transcription start sites (TSS) was mapped through intersecting the annotated genomic TSS locations (Zhang *et al*., 2022) with genomic coordinates of At‐RS31 iCLIP targets using bedtools. The most 5′ TSS for each gene was chosen with a custom R script, and the distribution of At‐RS31 binding site peaks up to 250 nt downstream of the TSS locations was plotted with the ggplot2 R package.

### Generation of glutathione S‐transferase‐tagged At‐RS31 constructs and protein purification for RNAcompete assay

Cloning and protein purification were performed as described previously (Ray *et al*., 2017). The full‐length At‐RS31 construct (*T7::GST‐31FL*) and RRMs‐only construct (*T7::GST‐31RRMs*), containing the first 175 amino acids, were generated from *A. thaliana* cDNA by PCR. An artificial stop codon was introduced by site‐directed PCR mutagenesis. The constructs were cloned into pTH6838 (Ray *et al*., 2013), downstream of the T7 promoter and fused *N*‐terminally to glutathione S‐transferase (GST; Fig. S3a). Primers are listed in Table S1.

Glutathione S‐transferase‐tagged proteins were expressed in *E. coli* BL21 cells grown at 37°C, lysed by sonication in phosphate‐buffered saline with protease inhibitor (Roche), and purified using glutathione sepharose 4B beads (GE Healthcare, Vienna, Austria). Proteins were eluted, and their concentration and size were assessed by spectrophotometry and SDS‐PAGE (Fig. S3b).

### RNAcompete assay

RNAcompete assay was performed using purified GST‐At‐RS31 and GST‐At‐RS31‐RRMs fusion proteins for pull‐down experiments. RNAcompete analysis was performed essentially as described in Ray *et al*. (2013, 2017).

### RNAcompete CAGA motif statistics

To compare the At‐RS31 motifs retrieved by RNAcompete with the iCLIP binding sites, the TAIR10 genome was scanned for positions matching the RNAcompete consensus motif CAGA using fimo v.4.11.1 (Grant *et al*., 2011). The RS31‐GFP binding site peaks were compared directly to the positions of CAGA sites with a custom R script.

### Plant material for RNA sequencing

We used three *A. thaliana* lines: the *At‐RS31* overexpression line: 35S::RS31, overexpressing the protein‐coding isoform AT3G61860.1 under the cauliflower mosaic virus (CaMV) 35S promoter; the *rs31‐1* mutant (SALK_021332, T‐DNA insertion in exon 5 of *At‐RS31*; Petrillo *et al*., 2014); and the WT Col‐0 (Fig. S1b–d). For each genotype, plants were grown in three biological replicates under the same conditions described for the iCLIP experiments.

### RNA isolation and RNA sequencing

Total RNA was isolated using the RNeasy Plant Mini Kit and treated with DNAse (both Qiagen). Strand‐specific transcriptome libraries were sequenced using 100 bp paired‐end reads on the Illumina Hi‐seq 2000 system (Next‐Generation Sequencing Facility, Vienna BioCenter) yielding 30–40 gigabases for each biological replicate.

### Transcript quantification

The transcript per million (TPM) expression was estimated with Salmon with the additional ‐‐gcBias and ‐‐validateMappings flags (v.0.14.1; Patro *et al*., 2017) for the Reference Transcript Dataset for Arabidopsis 2‐Quantification of Alternatively Spliced Isoforms (AtRTD2‐QUASI) annotation (Zhang *et al*., 2017).

### Read alignment with star

Reads were mapped to the index based on the TAIR10 genome release and the AtRTD2 transcriptome with star (v.2.7.1a; Dobin *et al*., 2013) using a 2‐pass mapping. The following parameters were used: ‐‐outSAMprimaryFlag AllBestScore, ‐‐outFilterMismatchNmax 2/0 (first/second pass), ‐‐outSjfilterCountTotalMin 10 5 5 5, ‐‐outFilterIntronMotifs RemoveNoncanonical, ‐‐alignIntronMin 60, ‐‐alignIntronMax 6000, ‐‐outSAMtype BAM SortedByCoordinate. During the second pass, the splice junction files of the control and test samples were passed to the mapping via the ‐‐sjdbFileChrStartEnd flag.

### Differential gene expression analysis

Differential expression (DE) analysis was performed using 3D RNA‐seq App (Guo *et al*., 2020). Read counts and TPM reads were generated using the tximport R package v.1.10.0 and the lengthScaledTPM method (Soneson *et al*., 2016) based on transcript quantifications from Salmon (Patro *et al*., 2017). Lowly expressed transcripts and genes were filtered by analysing mean–variance trend of the data. Transcripts were considered expressed if they had counts per million (CPM) ≥ 1 in at least three of the nine samples, providing an optimal filter for low expression. A gene was considered expressed if any of its transcripts met the above criteria. The TMM method was used to normalize gene and transcript read counts to log_2_‐CPM (Bullard *et al*., 2010). Limma R package was used for 3D expression comparison (Law *et al*., 2014; Ritchie *et al*., 2015). Expression changes were compared between *rs31‐1 vs* WT and 35S::RS31 vs WT contrast groups. For DE genes, the log_2_ fold change (L2FC) of gene abundance was calculated, and significance was determined using a *t*‐test. *P*‐values of multiple testing were adjusted for false discovery rate (Benjamini & Yekutieli, 2001). Genes were considered significantly differentially expressed if they had an adjusted *P*‐value < 0.05 and |L2FC| ≥ 1.

### Differential alternative splicing analysis

Alternative splicing events were obtained and quantified using whippet, v.0.11 (Sterne‐Weiler *et al*., 2018). Two separate splice graph indices were generated: one for ES, alternative acceptor (AA) and alternative donor (AD) events, and another for RIs and EIs. Both indices were based on the AtRTD2 transcriptome annotation (Zhang *et al*., 2017), supplemented with the STAR RNA‐seq alignments, and generated with the additional ‐‐bam‐min‐reads 10 flag. The RI/EI index was further supplemented with ‘pre‐mRNA’ gene coordinates and EI splice junctions detected using an in‐house script. ‘Pre‐mRNA’ coordinates ranged from the start to the end of genes, allowing quantification of the retention levels of all annotated introns in a gene. The Whippet delta step was run with default parameters, except for the ‐‐min‐samples 3 flag. Alternative acceptor and AD events were filtered to ensure both (alternative) junctions were detected in Whippet data. Retained intron events were required to have at least one read in either all control and/or test samples. Events with probability ≥ 0.9 and absolute delta percent spliced‐in (|ΔPSI|) ≥ 0.1 were considered significant differential alternative splicing (DAS) events.

### Functional enrichment analysis

Functional gene set enrichment analysis was performed using the g:GOSt tool of the g:Profiler (Kolberg *et al*., 2023; annotation version e110_eg57_p18_4b54a898) at https://biit.cs.ut.ee/gprofiler/gost.

### Statistical analysis of gene overlaps and Venn diagrams

The intersections between gene lists were calculated and visualized using the Venn diagram tool available at http://bioinformatics.psb.ugent.be/webtools/Venn/. The statistical significance of gene overlaps was assessed using a hypergeometric‐based tool available at http://nemates.org/MA/progs/overlap_stats.html. This tool calculates a representation factor, defined as the ratio of observed overlap to expected overlap and determines a *P*‐value using the hypergeometric (Fisher's exact) test or, for very large sets, a normal approximation.

### Phenotype analysis

Seeds were sown on ½MS with MES and 1% sucrose supplemented with none (0), 0.5, 1, or 2 μM abscisic acid (ABA). Following stratification for 3 d at 4°C in the dark, seeds were transferred to growth conditions of 21°C, 16 h : 8 h, light : dark, 80–100 μmol m^−2^ s^−1^ for 9 d. For the analysis of the cotyledon greening phenotype, three independent biological replicates were scored, each consisting of 25 seedlings per genotype. Cotyledons were assessed for both opening and greening. Statistical analyses were performed using two‐way ANOVA with ABA treatment (T) and genotype (G) as factors. Percentage data for greening were angularly transformed before analysis. Graphs were generated using graphpad prism v.9.00 for Windows (San Diego, CA, USA). When ABA treatment‐by‐genotype (T × G) interaction was significant (*P* < 0.05), Tukey's *post hoc* test was used to evaluate mean differences. For root growth analysis, seedlings were grown vertically, and primary root lengths were measured using the imagej software (http://rsweb.nih.gov/ij/). Six independent biological replicates were analysed, each including 8–15 seedlings per line. The experiments were repeated with two independent seed populations, yielding similar results. Statistical analyses were carried out using ANOVA (*P* < 0.05) and Tukey's *post hoc* test for comparisons.

### Reverse transcription polymerase chain reaction and alternative splicing analyses

Whole seedlings were harvested and ground in liquid nitrogen using a mortar and pestle. Total RNA was extracted using RNAeasy Plant Mini Kit (Qiagen) or TriPure Isolation Reagent (Roche). For cDNA synthesis, 1 μg of total RNA was reverse‐transcribed using either avian myeloblastosis virus or Moloney murine leukaemia virus reverse transcriptase with oligo(dT) primers, following the manufacturer's instructions. Polymerase chain reaction amplification was performed using Taq polymerase for 30–35 cycles. Primers are listed in Table S1. Alternative splicing patterns were analysed by gel electrophoresis. For a subset of genes, splicing isoforms were quantified by densitometry using the imagej software (http://rsweb.nih.gov/ij/). In these cases, a splicing index was calculated as the ratio of the longest isoform to the total signal of all the isoforms of the gene. Statistical analyses were performed using the infostat software (DiRienzo *et al*., 2018) (http://www.infostat.com.ar) with one‐way ANOVA and Fisher's LSD test for comparisons.

### Visualization of transcript isoform schematics

Transcript models for the genes are visualized using the Boxify tool (Riegler *et al*., 2021) available at https://boxify.boku.ac.at/.

## Results and Discussion

### Genome‐wide determination of At‐RS31 target transcripts and its binding motifs by iCLIP

To identify transcripts bound by At‐RS31 *in vivo* across the transcriptome, we utilized iCLIP (Meyer *et al*., 2017; Arribas‐Hernandez *et al*., 2021). For this purpose, we generated RS31::RS31‐GFP plants expressing At‐RS31 fused to GFP under the control of the endogenous promoter in the *rs31‐1* mutant background, as well as 35S::GFP control plants expressing GFP alone under the control of the 35S CaMV promoter (Fig. S2). The At‐RS31‐GFP fusion protein localised to the nucleus but was absent from the nucleolus, forming nuclear speckle‐like aggregates (Fig. S4), consistent with previous studies demonstrating At‐RS31 localization using antibodies against the native protein and At‐RS31‐GFP expressed under the 35S promoter (Docquier *et al*., 2004; Lorkovic *et al*., 2004a, 2008; Tillemans *et al*., 2005). To confirm the functionality of the RS31::RS31‐GFP fusion protein, we assessed its ability to complement the splicing defects observed in the *rs31‐1* mutant (Fig. S5). Reverse transcription polymerase chain reaction (RT‐PCR) analysis showed that its expression closely mirrors endogenous At‐RS31 in WT plants, both in terms of transcript levels and alternative splicing isoform ratios (Fig. S5a,b). Additionally, *RS31::RS31‐GFP* restored the splicing pattern of *At‐RS31* paralog *At‐RS31a*, which is disrupted in the *rs31‐1* mutant (Fig. S5c), further supporting the functional integrity of the fusion protein. These findings confirm that the At‐RS31‐GFP fusion protein is correctly expressed, localized, and retains its regulatory role in alternative splicing.

**Fig. 1:**
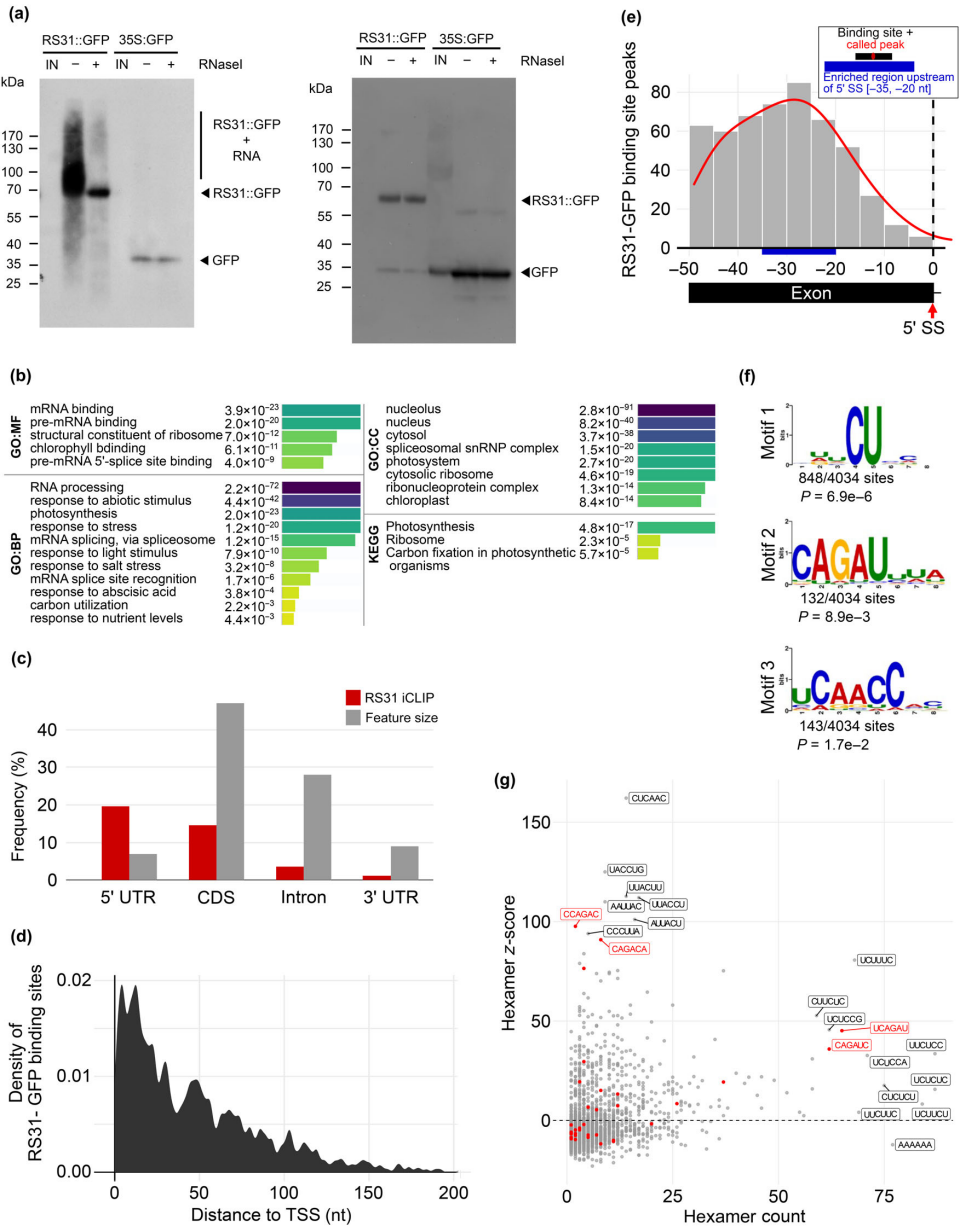
Determination of *Arabidopsis thaliana* serine/arginine‐rich (SR) protein At‐RS31 *in vivo* binding sites by individual‐nucleotide resolution crosslinking and immunoprecipitation (iCLIP). (a) Left: autoradiogram of RS31‐GFP and green fluorescent protein (GFP) protein–RNA complexes. After ultraviolet crosslinking, cell lysates were subjected to immunoprecipitation with GFP Trap beads. RNAs were radioactively labelled, and the complexes were separated by denaturing gel electrophoresis. IN, input (lysate). Treatment of the lysate with RNase I (+ RNase) indicates the size of the precipitated proteins. The region above the fusion protein containing the co‐precipitated RNAs used for library preparation is indicated. Right: iCLIP western blot. Immunoblot analysis of the membrane shown in the left panel with anti‐GFP antibody. Bands for GFP and RS31‐GFP are marked accordingly. (b) Functional profiling of genes containing At‐RS31 binding sites. Gene Ontology (GO) term enrichment analysis was performed using the g:GOSt tool from g:Profiler. Numeric and colour‐coded (capped at –log_10_ (*P*_adj) ≤ 16) *P*‐values are shown for the enriched GO terms and Kyoto Encyclopedia of Genes and Genomes (KEGG) pathways. BP, biological process; CC, cellular compartment; MF, molecular function. (c) Distribution of the At‐RS31 binding sites within protein‐coding transcripts. Distribution of RS31‐GFP binding sites within transcripts in relation to the total genomic length of the transcript features. 5′ UTR and 3′ UTR – 5′ and 3′ untranslated regions; CDS, coding sequences. (d) Distribution of the At‐RS31 binding sites within 5' UTRs relative to the transcription start sites (TSS). Distance to TSS is shown in nucleotides (nt). (e) Distribution of At‐RS31 binding sites peaking upstream of 5′ splice sites (5′ SS). Only exons at least 50 nt in length were analysed. The red line represents the local density of binding sites. The blue box marks the −35–20‐nt region upstream of 5′ SS where RS31‐GFP binding sites are enriched. (f) Significant STREME binding site motifs. Sequence logos of the most significant (based on their *P*‐value) RS31‐GFP binding motifs identified by STREME analysis. For the analysis, only sequences from the 9‐nt binding site regions were considered. (g) Hexamer counts and *z*‐scores. Scatterplot showing hexamer frequencies and counts computed from the 9‐nt binding site sequences of the RS31‐GFP iCLIP sample. Hexamer counts (*x*‐axis) are compared against hexamer *z*‐scores (*y*‐axis). The most enriched hexamers and the ones with highest counts are labelled according to their sequence. The highlighted hexamers (red) contain the subsequence CAGA. RS, arginine/serine.

To perform iCLIP, RS31::RS31‐GFP and 35S::GFP plants were subjected to UV crosslinking to preserve *in vivo* RNA–protein interactions. After cell lysis, the RNA–protein complexes were immunoprecipitated with GFP Trap beads, and the co‐precipitated RNAs were radiolabelled. Upon denaturing gel electrophoresis, membrane transfer, and autoradiography, RNA–protein complexes were detected in crosslinked RS31::RS31‐GFP plants (Fig. 1a, left). RNase I treatment strongly reduced the signal, indicating that indeed RNA was crosslinked. For plants expressing GFP alone, only little RNA was crosslinked. The identity of the proteins was confirmed by probing the membrane with an anti‐GFP antibody (Fig. 1a, right). The membrane region corresponding to the covalently linked RS31‐GFP–RNA complexes was excised (Fig. S6a). Similarly, we excised the region above the GFP band (Fig. S6b). RNA was isolated for the preparation of libraries for high‐throughput sequencing from three biological replicates (Fig. S6c,d). The read statistics are presented in Table S2. As reverse transcriptase stops at the remnants of the digested protein, the binding sites can be retrieved with single nucleotide resolution. A global view of the localization of the crosslink sites of RS31‐GFP and GFP alone throughout the chromosomes is shown in Fig. S7.

PureCLIP was used to call peaks from the iCLIP reads (Krakau *et al*., 2017). For RS31‐GFP and GFP, 6939 and 508 peaks were recovered, respectively (Table S2). If peaks were directly adjacent, we considered only the one with the highest score assigned by PureCLIP. Peaks were then extended by four nucleotides in both directions to define binding sites of nine nucleotides. This resulted in 4236 binding sites for RS31‐GFP and 324 binding sites for the GFP control. Of the 324 GFP‐only binding sites, 202 were located within nine nucleotides of RS31‐GFP binding sites and were subtracted, leaving 4034 RS31‐GFP binding sites (Tables S2, S3) assigned to 1142 protein‐coding and 279 noncoding genes (Table S4).

To gain insights into the At‐RS31 targets and the molecular processes and biological pathways it may influence, we performed a functional enrichment analysis of the 1421 iCLIP target genes using g:Profiler (Kolberg *et al*., 2023; Fig. 1b; Table S5). The analysis revealed a significant enrichment of genes involved in various stress responses, as well as in photosynthesis, carbon utilization, and plant responses to light, supporting our previous findings on the regulation of *At‐RS31* alternative splicing by chloroplast retrograde signalling and TOR kinase in response to photosynthesized sugars and light (Petrillo *et al*., 2014; Riegler *et al*., 2021). Furthermore, At‐RS31 targets are associated with RNA splicing, particularly mRNA splicing via the spliceosome, underscoring its role in co‐ and/or post‐transcriptional regulation (Table S5).

Out of the 1142 protein‐coding genes containing At‐RS31 binding sites, 61.3% (700) have them in the 5′ UTR, 43.2% (493) in the coding region, 11.7% (134) in introns, and 4% (46) in the 3′ UTR (Fig. 1c). Additionally, there is an accumulation of At‐RS31 binding sites towards the transcriptional start site in many genes (Fig. 1d; Table S6), a pattern previously observed for putative *cis*‐elements of the Arabidopsis SR‐like protein SR45 (Xing *et al*., 2015). The functional significance of this distribution remains to be determined.

Since At‐RS31 is a splicing regulator that interacts with the U1‐70K and U11/U12‐31 K proteins, which are involved in the recognition of the 5′ splice site (Lorkovic *et al*., 2005; Altmann *et al*., 2020), we determined the position of the At‐RS31 binding site peaks (centre position of binding site) relative to 5′ splice sites (Fig. 1e). For this, only exons with a length of at least 50 nt were considered. The distribution of binding site peaks has a slight enrichment at 25–30 nt upstream of the 5′ splice sites. Therefore, we asked whether these sites share conserved sequence motifs. Binding site peaks located 25 to 30 nt upstream of 5′ splice sites were extracted, extended to 21 nt, and aligned (Fig. 1e; Table S7). The alignment of the sequences consists mainly of C and U and is presented as a sequence logo in Fig. S8. Therefore, the polypyrimidine‐rich sequences may impact the regulation of 5′ splice site choice by attracting At‐RS31.

Investigating 4034 At‐RS31 binding sequences using STREME revealed three enriched motifs (Fig. 1f). The most significant pattern, Motif 1, features a CU sequence, found at 848 binding sites (21%). The second most significant motif, Motif 2 (CAGAU), occurs in 132 sites (3.3%), and Motif 3, UCAACC, is present in 143 sites (3.5%).

In parallel, we searched for hexamer motifs enriched within the binding sites. Among the motifs with the highest *z*‐scored hexamers, predominantly CU and AC combinations are enriched (Fig. 1g). CU combinations (polypyrimidines) appear among the most frequent but less enriched hexamers. In addition, the motif CAGA is present among the enriched and highest counts (highlighted in red, Fig. 1g).

### Consistent and divergent RNA‐binding motifs identified by RNAcompete and iCLIP for At‐RS31

**Fig. 2:**
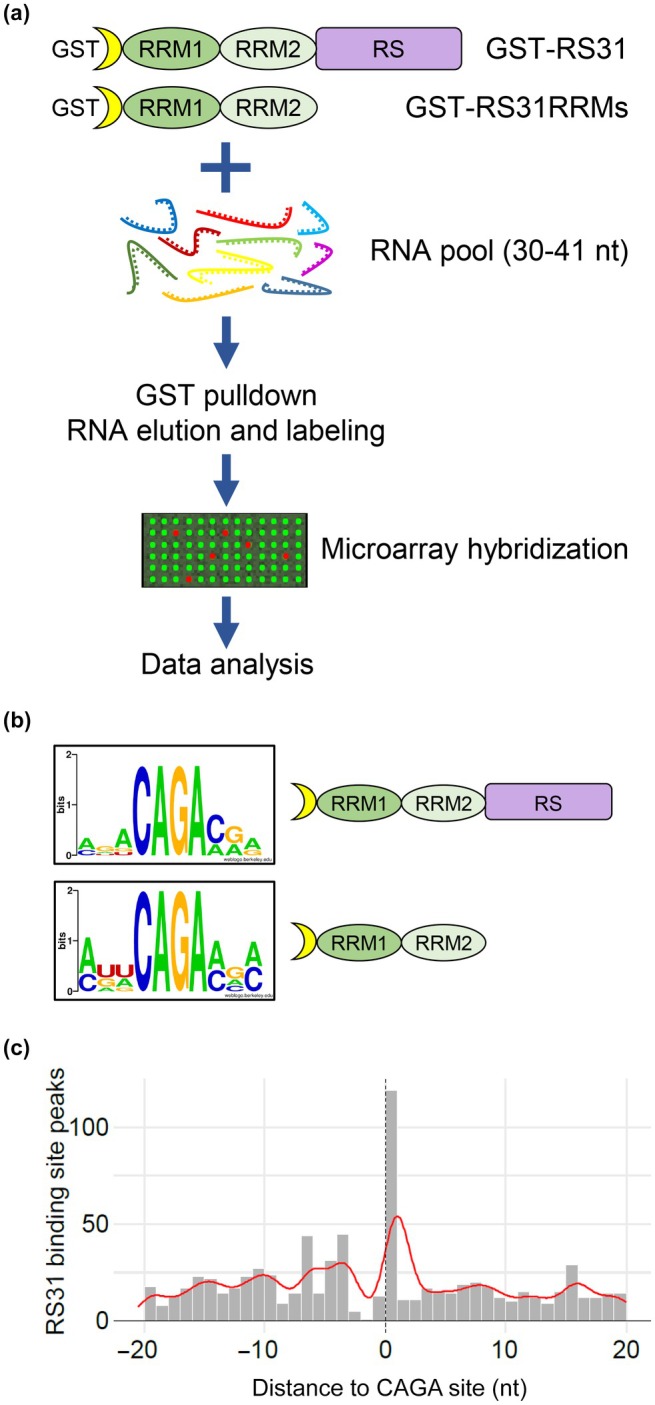
Identification of *Arabidopsis thaliana* Serine/Arginine‐rich (SR) protein At‐RS31 RNA‐binding motifs using RNAcompete. (a) Overview of the RNAcompete assay. Glutathione S‐transferase (GST)‐tagged full‐length At‐RS31 protein and its truncated version comprising both RNA recognition motifs (RRMs) were incubated with a 75‐fold molar excess of designed RNA pool. RNA bound to GST‐RS31 and GST‐RS31RRMs fusion proteins during the GST pulldown was eluted, purified, labelled, and hybridized to custom Agilent 244 K microarrays. Microarray data were analysed computationally to identify 7‐mers specifically bound by At‐RS31 and generate RNA‐binding motifs. RRM1 and RRM2, RNA recognition motif domains; RS, region rich in arginines and serines; nt, nucleotides. (b) RNA‐binding motifs of the full‐length At‐RS31 and its truncated version containing RRMs identified in the RNAcompete assay and represented as sequence logos. (c) Individual‐nucleotide resolution crosslinking and immunoprecipitation (iCLIP) binding site distance to RNAcompete motif sites. Distribution of RS31‐GFP binding site peaks in relation to CAGA sites across At‐RS31 target transcripts. The position 1 on the *x*‐axis denotes the C in the CAGA motif identified by the RNAcompete assay. RS, arginine/serine.

To validate the At‐RS31 *in vivo* binding sites obtained through iCLIP, we employed RNAcompete that challenges RNA‐binding proteins (RBPs) with a pool of short RNAs (30–41 nt) that include almost every possible 7‐mer sequence combination (Fig. 2a; Ray *et al*., 2013; Ray *et al*., 2017). For this, we generated constructs harbouring full‐length At‐RS31 and its truncated version with the two RRMs but lacking the RS region, respectively (Fig. S3a). The corresponding GST fusion proteins, GST‐31 FL and GST‐31 RRMs, were purified from *E. coli*, and their integrity was verified (Fig. S3b).

The RNAcompete experiments revealed AGACAGA as the highest scoring 7‐mer (Fig. S9). Intriguingly, the top 10 motifs bound by GST‐31 FL and GST‐31 RRMs contain a core 4‐mer CAGA (Figs 2b, S9), suggesting that both variants have very similar *in vitro* binding properties and that the RS region does not contribute significantly to At‐RS31 binding specificity *in vitro*. The CAGA 4‐mer was also present at the At‐RS31 binding sites identified by iCLIP (Motif 2, Fig. 1f) and within high‐scoring hexamers (Fig. 1g).

We further analysed the distance of the At‐RS31 iCLIP binding site peaks relative to the CAGA motif (Fig. 2c). The results revealed a positive correlation between the binding sites and the CAGA motif, with a higher number of binding site peaks positioned towards the cytosine (C) of the CAGA motif.

Overall, the RNA‐binding motifs identified by RNAcompete for At‐RS31 are highly consistent with the motifs identified by iCLIP, although iCLIP has identified additional motifs (Fig. 1f). This difference may be attributed to the fact that iCLIP reports on binding in the native environment and thus also reflects post‐translational modifications and protein–protein interactions of At‐RS31 *in planta*. For example, differences between *in vitro* and *in vivo* binding recently have been systematically analysed in mammalian cells by comparing iCLIP with a newly developed *in vitro* iCLIP approach. *In vitro* iCLIP revealed the intrinsic binding properties of U2AF2, whereas iCLIP revealed the dependence of binding of U2AF2 on interactions with a suite of other proteins *in vivo* (Sutandy *et al*., 2018). Hence, the combination of both *in vitro* (RNAcompete) and *in vivo* (iCLIP) methods provides a more comprehensive understanding of the RNA‐binding preferences of At‐RS31 and highlights the importance of considering the context in which RBPs function.

### At‐RS31 shapes alternative splicing patterns and gene expression in Arabidopsis

To better understand the biological processes influenced by At‐RS31 and its role in regulating gene expression, we performed a transcriptome analysis using RNA‐seq on the *rs31‐1* mutant, plants overexpressing the mRNA1 protein‐coding isoform (*35S::RS31*), and WT control plants (Fig. S1b–d). Under normal growth conditions, 35S::RS31 and *rs31‐1* plants show only minor phenotypic differences from WT plants (Fig. S1d), with 35S::RS31 plants being slightly smaller, as described previously (Petrillo *et al*., 2014).

We identified 442 DAS events across 381 genes in *rs31‐1* plants, and 2949 DAS events across 2063 genes in 35S::RS31 plants, using |ΔPSI| ≥ 0.1 as a threshold (Table S8). Interestingly, 42.5% (162 out of 381) of the genes affected by DAS in *rs31‐1* plants were also affected in 35S::RS31 plants. However, only *c*. 30% (130 out of 442) of the specific DAS events were shared between the two lines (Table S8), suggesting that alterations in At‐RS31 levels lead to distinct splicing outcomes within the same genes, depending on whether At‐RS31 is underexpressed or overexpressed.

**Fig. 3:**
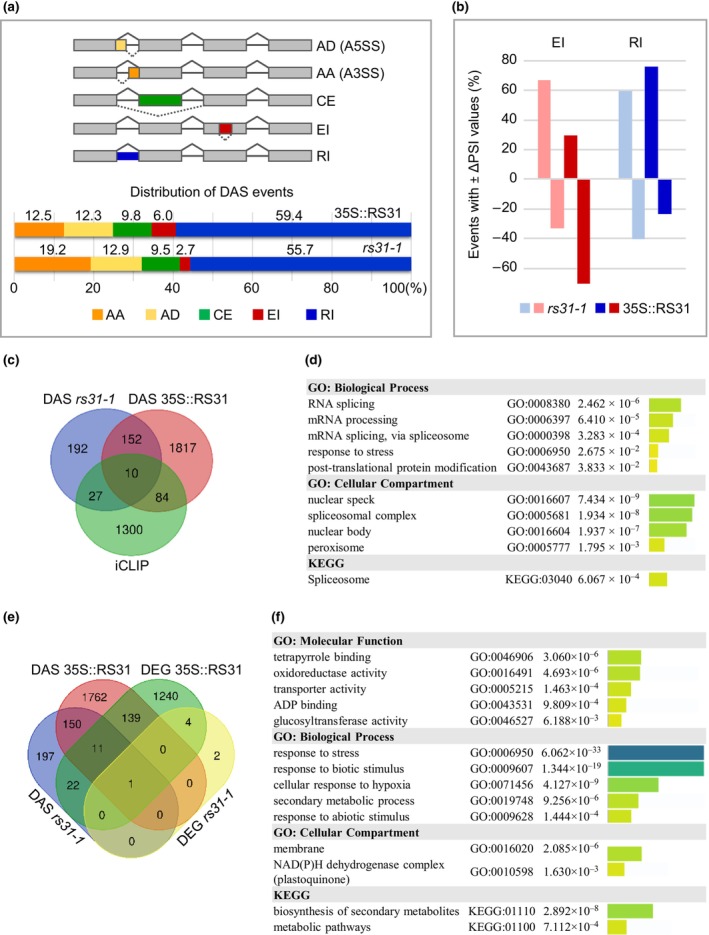
Impact of At‐RS31 on alternative splicing and gene expression in *Arabidopsis thaliana*. (a) Distribution of alternative splicing event types differentially regulated in *rs31‐1* and 35S::RS31 lines in comparison with wild‐type (WT) *A. thaliana*. Diagrams on the top illustrate the analysed alternative splicing event types: alternative donor (AD, or alternative 5′ splice site), alternative acceptor (AA, or alternative 3′ splice site), cassette exon (CE), exitron (EI), and retained intron (RI). (b) Proportions of differential EI and RI events with reduced or increased splicing efficiency in *rs31‐1* and 35S::RS31 lines compared with WT. Positive and negative percentages indicate the proportion of events with increased or decreased per cent spliced‐in values (ΔPSI), respectively. (c, e) Venn diagram comparisons of genes exhibiting At‐RS31 individual‐nucleotide resolution crosslinking and immunoprecipitation (iCLIP) binding sites, differential alternative splicing (DAS), and differential expression (DE) in *rs31‐1* and 35S::RS31 relative to WT. (c) Overlap between DAS genes and genes with At‐RS31 iCLIP binding sites. (e) Overlap between DAS and DE genes. (d, f) Functional profiling of (d) DAS genes containing At‐RS31 binding sites and (f) 35S::RS31 DE genes. The Gene Ontology (GO) term enrichment analysis was performed using the g:GOSt tool of the g:Profiler. Numeric and colour‐coded (capped at –log_10_(*P*_adj) ≤ 16) *P*‐values are shown for the enriched GO terms and Kyoto Encyclopedia of Genes and Genomes (KEGG) pathways. RS, arginine/serine.

Previous studies in plants have consistently reported that alternative 3′ splice sites (AA) are generally used about twice as frequently as alternative 5′ splice sites (AD; Wang & Brendel, 2006; Marquez *et al*., 2012; Zhang *et al*., 2022). This ratio is nearly constant across species in different kingdoms (McGuire *et al*., 2008). However, in *rs31‐1* and 35S::RS31 plants, the AA : AD ratio was reduced to *c*. 1.4 : 1 and *c*. 1 : 1, respectively (Fig. 3a; Table S8), suggesting that At‐RS31 may influence this balance. One explanation could be that At‐RS31 modulates the recognition and usage of a subset of 5′ splice sites, as implied by the distribution of binding site peaks 25 to 30 nt upstream of 5′ splice sites (Fig. 1e).

Retained intron was the most frequent DAS event type in both *rs31‐1* (55.7%) and 35S::RS31 (59.4%) plants (Fig. 3a), a higher prevalence than the *c*. 40% reported for WT plants (Marquez *et al*., 2012). We have previously shown that RIs and EIs, defined as alternatively spliced internal regions of protein‐coding exons, have distinguishable features, including their regulation and functional outcomes (Marquez *et al*., 2015; Jabre *et al*., 2021). Indeed, the direction of splicing changes for RIs and EIs was significantly different (*P*‐value 7.51 × 10^−39^) in 35S::RS31 plants, where retention of introns was predominantly promoted (76.2%), while splicing of EIs was predominantly enhanced (70.2%; Fig. 3b). Conversely, in *rs31‐1* plants, splicing of EI was inhibited in 66.7% of DAS EIs (Table S8). These contrasting patterns of EI splicing between *rs31‐1* and 35S::RS31 plants suggest a positive role for At‐RS31 in promoting EI splicing.

To assess the magnitude of these splicing shifts, we also examined DAS events displaying large amplitude changes (|ΔPSI| ≥ 0.3). In *35S::RS31*, EIs showed the highest fraction of such strongly shifted events (37.6%), followed by RIs (19.8%), while other event types accounted for only 11.9–14.6% of such events (Table S8). Moreover, EIs were more frequently shifted in the negative direction (ΔPSI ≤ −0.3) than positive (ΔPSI ≥ 0.3), at 29.2% vs 8.4%, whereas RIs showed the opposite trend (4.6% negative vs 15.1% positive). These findings illustrate that *At‐RS31* overexpression not only confers different directions of regulation for EIs and RIs but also tends to drive large splicing changes for these event types.

We could not reliably assess the inhibition of EI splicing and intron retention in *rs31‐1* under the strong‐change criterion (|ΔPSI| ≥ 0.3) because, as described previously (Marquez *et al*., 2012, 2015), EI isoforms in WT typically show PSI ≥ 0.9 whereas RIs generally have PSI ≤ 0.1. These extreme baseline values leave little room for further measurable changes in *rs31‐1*, even if EI splicing or intron retention is suppressed. Consequently, regulatory shifts in *rs31‐1* may remain underestimated.

The comparison between DAS and iCLIP data sets revealed that At‐RS31 binds to the transcripts of 37 and 94 DAS genes in *rs31‐1* and 35S::RS31, respectively (Fig. 3c; Table S8). Differential alternative splicing events in selected genes with At‐RS31 binding sites were validated using RT‐PCR (Fig. S10). Among the 10 genes presenting overlap between the three data sets (Fig. 3c), we identified *GOX1*, involved in the photorespiratory pathway (Kerchev *et al*., 2016); the senescence‐associated gene *SEN1* (Oh *et al*., 1996); the RING‐type ubiquitin E3 ligase *AtCHYR2*, functioning in glucose and ABA signalling (Wang *et al*., 2023); *NCH1*, essential for the phototropin‐mediated chloroplast accumulation response (Suetsugu *et al*., 2016); and four RBPs – *At‐RS31* itself, its paralogues *At‐RS31a* and *At‐RS40* (Lopato *et al*., 1996; Kalyna & Barta, 2004), and the glycine‐rich protein *AtGRP8* (Streitner *et al*., 2012). Overall, Gene Ontology (GO) term analysis showed that DAS genes with At‐RS31 iCLIP binding sites were associated with terms related to mRNA splicing via spliceosome, spliceosomal complex, and nuclear speck (Fig. 3d; Table S5), further underscoring the role of At‐RS31 in alternative splicing regulation and its integration into the splicing machinery.

The impact of At‐RS31 on alternative splicing was more pronounced than its effect on gene expression (Fig. 3e). Overexpression of At‐RS31 resulted in the DE of 1417 genes, with 798 up‐ and 619 downregulated genes (|Log_2_FC| > 1; Table S9). Only six DEGs were identified in the *rs31‐1* mutant, excluding *At‐RS31* itself. Notably, 7.3% (151 out of 2063) of 35S::RS31 DAS genes were also differentially expressed (Fig. 3e), indicating largely separate effects of At‐RS31 on alternative splicing and gene expression.

Cross‐referencing the DE and iCLIP data showed that At‐RS31 binds to the transcripts of 77 35S::RS31 DEGs (Table S9). The balanced distribution between upregulated (41) and downregulated (36) genes with At‐RS31 binding sites suggests that At‐RS31 can have both positive and negative effects on gene expression. However, it is possible that much of the DE observed may be downstream consequences of At‐RS31's direct regulatory function on splicing.

The impact of At‐RS31 on transcription factor (TF) splicing may account for some of the observed DE via alternative TF isoforms with altered regulatory properties. Out of 2534 TFs (Calixto *et al*., 2018), At‐RS31 binding sites were found on the transcripts of 107 TFs, while 192 and 39 TFs showed DAS in 35S::RS31 and *rs31‐1* plants, respectively (Tables S8; TF column, and S10). For example, At‐RS31 binds to *WRI4/AP2‐2* (AT1G79700), which responds to ABA, light, and carbohydrate availability (Vogel *et al*., 2012). It promotes two in‐frame DAS events (AD and CE removing 10 and 47 amino acids, respectively) that affect the AP2 DNA‐binding domain and may influence its regulatory function. Similarly, At‐RS31 modulates *HMGB2* (AT1G20693), a TF involved in stress responses to cold, drought, and salt stress (Kwak *et al*., 2007). It promotes an AD event in exon 3, introducing a PTC (Table S8; Fig. S10), likely leading to NMD, as supported by the observed downregulation of *HMGB2* expression levels (Table S9). These splicing changes may influence TF regulatory functions and lead to alterations in the expression of their target genes.

Finally, GO term enrichment of DEGs in the 35S::RS31 plants revealed a strong association with responses to stress, including hypoxia, and responses to both abiotic and biotic stimuli (Fig. 3f; Table S5). This contrasts with the enrichment patterns observed in DAS gene sets, corroborating the small overlap between DAS and DE genes (Fig. 3e) and emphasizing the separate mechanisms by which At‐RS31 influences alternative splicing and gene expression.

### At‐RS31 has a broad impact on RNA‐binding proteins and splicing factors

Gene Ontology term analysis revealed an enrichment in splicing and mRNA processing terms in the At‐RS31 iCLIP and DAS gene data sets (Table S5; Figs 1b, 3d). Moreover, the low overlap between iCLIP peaks and At‐RS31‐affected genes suggested that the regulatory impact of At‐RS31 could partly be indirect, likely by modulating other splicing factors (SFs). To investigate this, we examined the effects of At‐RS31 on alternative splicing of 798 known SFs and RBPs (Calixto *et al*., 2018). One hundred eighty‐four DAS events were identified in 117 SF/RBP genes in 35S::RS31 plants, and 27 DAS events were found in 17 SF/RBP genes in *rs31‐1* plants (Table S8; SF/RBP column). By contrast, DE was observed in far fewer SF/RBPs, with 13 DEGs in 35S::RS31 and none in *rs31‐1* (Table S9; SF/RBP column).

Reverse transcription polymerase chain reaction validation confirmed the DAS events in selected SF/RBPs (Fig. S11). Interestingly, At‐RS31 regulates EI splicing in some of these genes. For example, At‐RS31 inhibits splicing of an in‐frame EI in the flowering regulator *FPA*, which controls mRNA 3′ end formation (Hornyik *et al*., 2010), thereby favouring production of the full‐length protein (Fig. S11; Table S8). Similarly, the inhibition of out‐of‐frame EI splicing results in the production of full‐length proteins, as observed in the snRNA transcription activator *SRD2* (Ohtani & Sugiyama, 2005), the SF *RDM16*, required for RNA‐directed DNA methylation (Huang *et al*., 2013), the mRNA export factor *SAC3a* (Kanno *et al*., 2018), and the cold‐regulated kinase *CDKG1* (Cavallari *et al*., 2018).

Additionally, At‐RS31 binding sites were identified in 68 SF/RBPs (Table S4). Of these, 17 underwent DAS in 35S::RS31 plants, and three were affected in both *rs31‐1* and 35S::RS31 (Table S8). Besides SR proteins, this group includes CCR1/At‐GRP8, an hnRNP‐like glycine‐rich RBP (Streitner *et al*., 2012), CypRS64, a cyclophilin that interacts with SR proteins and components of U1 and U11 snRNPs (Lorkovic *et al*., 2004b), and CIS1, a SF involved in blue‐light‐mediated flowering via interaction with cryptochrome CRY2 (Zhao *et al*., 2022). The presence of At‐RS31 binding sites in these and other SF/RBPs suggests that they are likely direct targets of At‐RS31, positioning them as key players in its regulatory network.

### Cross‐regulation of SR protein family by At‐RS31

**Fig. 4:**
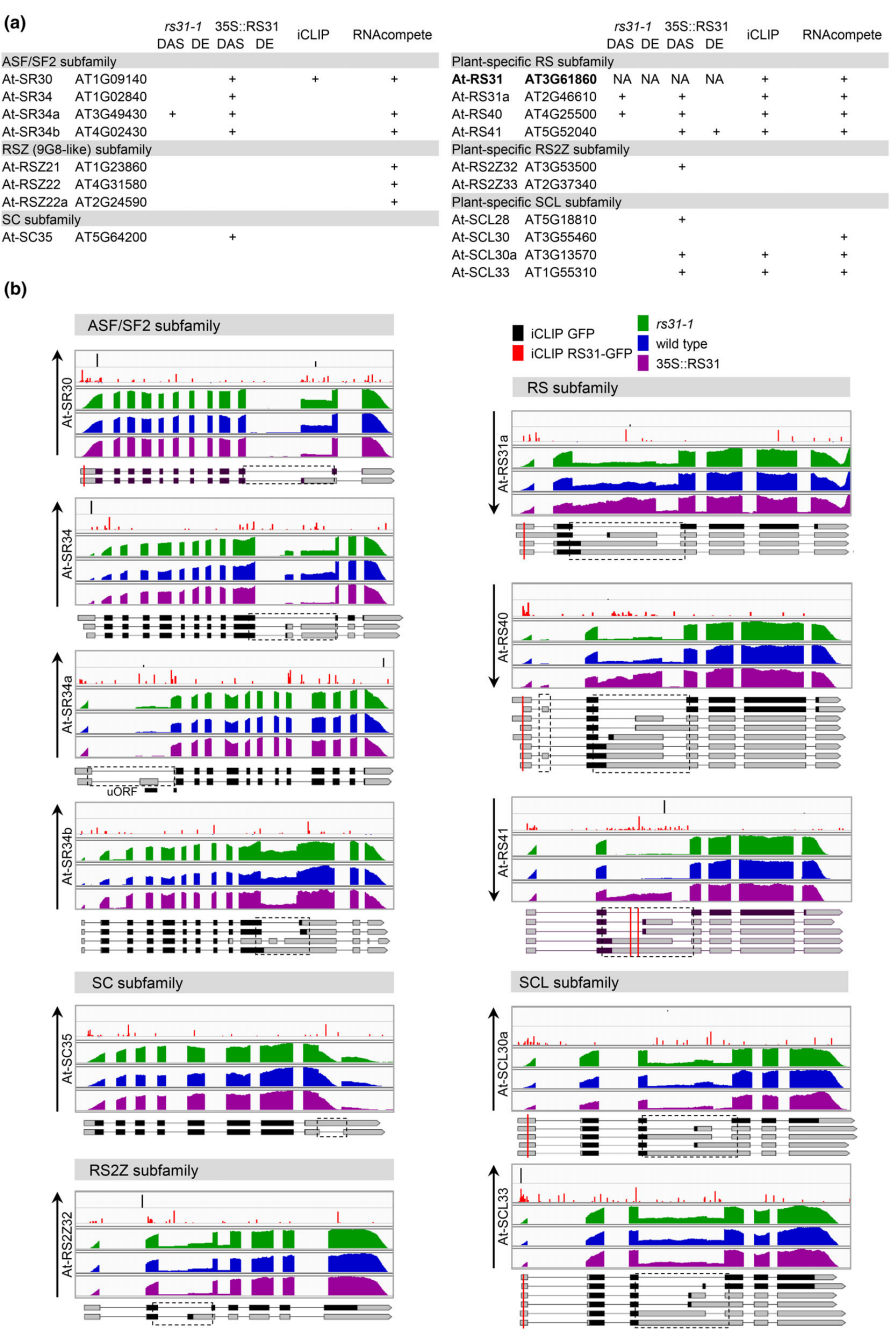
Regulation of Arabidopsis serine/arginine‐rich (SR) protein family by At‐RS31. (a) Overview of differential alternative splicing (DAS) and differential gene expression (DE) in Arabidopsis SR genes in *rs31‐1* mutant and 35S::RS31 overexpression plants, relative to wild‐type (WT) controls. The table includes information on individual‐nucleotide resolution crosslinking and immunoprecipitation (iCLIP) peaks and the top 7‐mers identified by RNAcompete within SR genes. ‘NA’ indicates data not applicable. (b) Integrated Genomics Viewer tracks depicting iCLIP crosslink sites for green fluorescent protein (GFP) and RS31‐GFP, along with RNA sequencing read coverage tracks for *rs31‐1*, 35S::RS31, and WT plants. The related tracks have the same scale. Transcript models for SR genes are displayed using the Boxify tool (https://boxify.boku.ac.at/). Protein‐coding regions, spanning from the translational start codon to the stop codon or premature termination codon, are shown in black. Red vertical lines represent the locations of At‐RS31 binding sites identified by iCLIP. Only transcripts relevant to the identified DAS events are displayed, with dashed rectangles marking these alternatively spliced regions. A black line denotes the position of an upstream open reading frame (uORF) in *At‐SR34a*. Black upward and downward arrows indicate an increase or a decrease, respectively, in the ratio of splice variants encoding full‐length SR proteins. RS, arginine/serine.

Serine/arginine‐rich proteins are regulated by alternative splicing, which involves both auto‐regulation and cross‐regulation within the SR protein family (Lopato *et al*., 1999; Sureau *et al*., 2001; Kalyna *et al*., 2003, 2006; Palusa *et al*., 2007; Müller‐McNicoll *et al*., 2019). Consistent with this, previous studies have shown that *At‐RS31* alternative splicing is influenced by other SR proteins, including At‐SR30, At‐RS2Z33, and the SR‐like protein SR45 (Lopato *et al*., 1999; Kalyna *et al*., 2006; Ali *et al*., 2007; Simpson *et al*., 2012; Carvalho *et al*., 2016). Here, we extend this network of cross‐regulation by identifying alternative splicing changes in two *At‐RS31* paralogs, *At‐RS31a* and *At‐RS40*, as well as the ASF/SF2 subfamily member *At‐SR34a* (Barta *et al*., 2010) in the *rs31‐1* mutant. Additionally, in the 35S::RS31 plants, 12 of the 18 Arabidopsis SR genes demonstrate DAS, with *At‐RS41* also showing DE (Fig. 4a; Tables S8, S9). The iCLIP analysis revealed At‐RS31 binding peaks in *At‐RS31a, At‐RS40*, *At‐RS41, At‐SR30, At‐SCL30a*, and *At‐SCL33*, suggesting their regulatory significance (Fig. 4; Tables S4, S8). Additionally, 13 SR genes contain top 7‐mers identified through RNAcompete (Fig. 4a).

All ASF/SF2 subfamily members (*At‐SR30, At‐SR34, At‐SR34a*, and *At‐SR34b*) display DAS events influenced by At‐RS31 (Fig. 4; Table S8). A notable example is CE skipping in the 5′ UTR of *At‐SR34a*, promoted in 35S::RS31 plants but inhibited in *rs31‐1* mutant. CE inclusion creates an upstream open reading frame (uORF), overlapping the primary ORF start site, leading the transcript to NMD (Kalyna *et al*., 2012). Thus, At‐RS31 likely upregulates At‐SR34a by controlling AS to counteract NMD. Overexpression of At‐RS31 also favours the protein‐coding isoforms of *At‐SR30, At‐SR34*, and *At‐SR34b* (Fig. 4b; Table S8). Therefore, At‐RS31 regulates AS in this subfamily, enhancing protein‐coding isoforms without changing overall gene expression.

In the RS2Z, SCL, and SC subfamilies, At‐RS31 also reduces unproductive AS isoforms, thus increasing the proportion of protein‐coding isoforms (Fig. 4b; Table S8). RS2Z and SCL genes contain remarkably long introns located between RNP2 and RNP1 motifs of their *N*‐terminal RRMs. These introns undergo highly conserved AS from the moss *Physcomitrium patens* to Arabidopsis, suggesting their critical regulatory roles (Kalyna & Barta, 2004; Iida & Go, 2006; Kalyna *et al*., 2006). For example, in *At‐RS2Z32*, At‐RS31 inhibits the usage of a conserved alternative 3′ splice site (Fig. 4b; Table S8). Similar to the AS events regulated by At‐RS31 in the ASF/SF2 genes, the DAS events in *At‐RS2Z32, At‐SCL30a*, and *At‐SCL33* (Fig. 4b; Table S8) predominantly result in AS transcripts sensitive to NMD (Palusa & Reddy, 2010; Kalyna *et al*., 2012). Interestingly, in *At‐SC35*, At‐RS31 inhibits splicing of an intron within the 3′ UTR, preventing NMD and promoting the accumulation of the protein‐coding variant (Palusa & Reddy, 2010; Fig. 4b; Table S8). A similar regulatory mechanism was found in the overexpression of SC35 in human cells (Sureau *et al*., 2001).

Conversely, in the plant‐specific RS subfamily, which includes *At‐RS31* and its paralogs *At‐RS31a*, *At‐RS40*, and *At‐RS41*, At‐RS31 favours unproductive AS variants, reducing the proportion of protein‐coding isoforms (Figs 4b, S11; Table S8). The AS in these genes also occurs in their longest introns and is conserved from Arabidopsis to the single‐celled green alga *Chlamydomonas* (Kalyna *et al*., 2006). The iCLIP analysis shows an accumulation of RS31‐GFP peaks within these introns, suggesting that At‐RS31 directly interacts with these regions (Fig. 4b). *At‐RS31a* and *At‐RS40* show opposite splicing regulation in *rs31‐1* and 35S::RS31 contexts. At‐RS31 promotes a shift towards CE‐containing NMD‐sensitive transcripts (Kalyna *et al*., 2012; Fuchs *et al*., 2021), decreasing the proportion of protein‐coding transcripts. *At‐RS41* follows a similar pattern but only in 35S::RS31 plants (Figs 4b, S11; Table S8). Overall expression of *At‐RS40* and *At‐RS41* is reduced in 35S::RS31 plants (Table S8). This demonstrates negative regulation by At‐RS31 of its paralogs through alternative splicing coupled to NMD, reducing the amount of their functional proteins. Similar cross‐regulatory mechanisms were described for the paralogs of Arabidopsis glycine‐rich proteins and polypyrimidine tract‐binding proteins (Schöning *et al*., 2007; Schöning *et al*., 2008; Stauffer *et al*., 2010; Burgardt *et al*., 2024). These mechanisms also occur in other taxa, where SR proteins and hnRNPs in mammals and Drosophila regulate each other's expression through alternative splicing coupled with NMD, maintaining splicing homeostasis (Jumaa & Nielsen, 1997; Kumar & Lopez, 2005; Rossbach *et al*., 2009).

Taken altogether, At‐RS31 increases the abundance of protein‐coding isoforms for most SR genes, except for its paralogs, where it promotes the accumulation of nonproductive transcripts. At‐RS31 serves as a general regulator of protein‐coding isoforms across different subfamilies of SR proteins in Arabidopsis, acting as a hierarchical regulator of alternative splicing.

### At‐RS31 and TOR pathway share targets regulating key aspects of plant growth and stress response

**Fig. 5:**
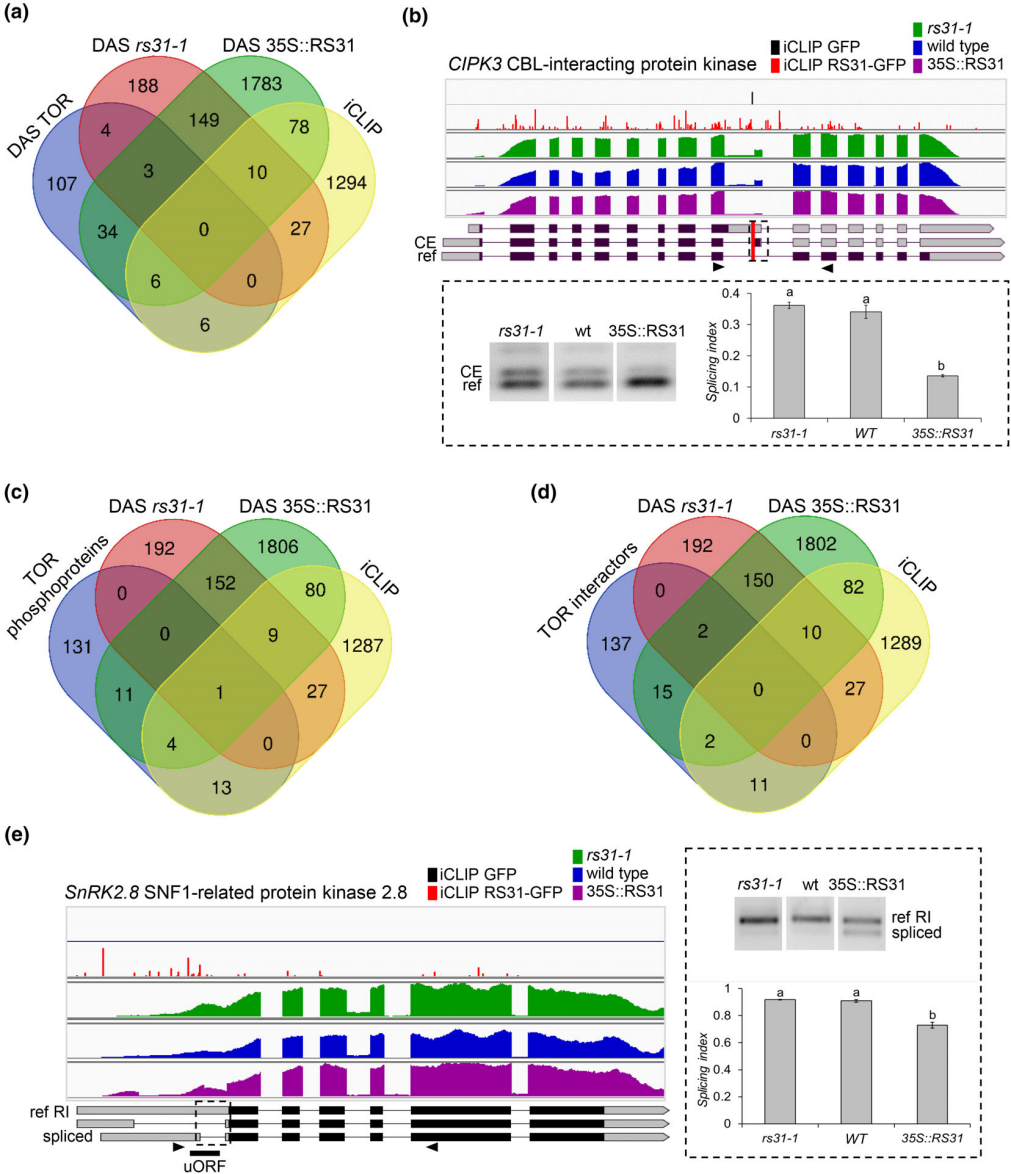
*Arabidopsis thaliana* serine/arginine‐rich (SR) protein At‐RS31 regulates alternative splicing of Target of Rapamycin (TOR)‐related genes. (a, c, d) Venn diagram comparisons showing the overlap between genes undergoing differential alternative splicing (DAS) in *rs31‐1* or 35S::RS31, genes with At‐RS31 binding sites identified by individual‐nucleotide resolution crosslinking and immunoprecipitation (iCLIP), and TOR‐related genes. (a) Genes undergoing DAS in response to TOR inhibition; (c) genes encoding TOR‐phosphorylated proteins; (d) genes encoding proteins interacting with TORC1 components. (b, e) At‐RS31 modulates alternative splicing of *CBL‐INTERACTING PROTEIN KINASE 3 (CIPK3*) (b) and *SNF1‐RELATED PROTEIN KINASE 2–8 (SnRK2.8*) (e). Integrated Genomics Viewer tracks show iCLIP crosslink sites for green fluorescent protein (GFP) and RS31‐GFP, alongside RNA sequencing read coverage for *rs31‐1*, 35S::RS31, and wild‐type (WT) plants. The related tracks have the same scale. In transcript models, protein‐coding regions (from the translational start codon to the stop codon or premature termination codon) are shown in black. Dashed rectangles highlight regions undergoing DAS. A red vertical line marks the At‐RS31 dominant binding site in *CIPK3*. A black line indicates the position of an upstream open reading frame (uORF) in *SnRK2.8*. (b) Lower panel and (e) right panel show representative reverse transcriptase polymerase chain reaction (RT‐PCR) gel images of *CIPK3* (b) and *SnRK2.8* (e) splicing patterns in *rs31‐1*, 35S::RS31, and WT. Arrow heads indicate primer positions. CE, cassette exon; ref, reference transcript; RI, retained intron. Bar graphs show splicing index values quantified using imagej. Bars represent mean ± SE (*n* = 3). Different letters indicate statistically significant differences between genotypes (*P* < 0.05 by ANOVA followed by Fisher's LSD test for multiple comparisons). RS, arginine/serine.

The TOR signalling pathway influences cell growth and metabolism in response to nutrients, growth factors, and environmental signals (Burkart & Brandizzi, 2021). Our previous research showed that TOR kinase regulates alternative splicing of *At‐RS31* in response to sugars and light, increasing the proportion of the protein‐coding splice variant (Riegler *et al*., 2021). To determine whether At‐RS31 target genes may be regulated through the crosstalk with the TOR pathway, we analysed 160 genes undergoing DAS in response to TOR activity modulation by glucose and the TOR inhibitor Torin2 (Riegler *et al*., 2021; Fig. 5a; Tables S11, S12). Of these, 7 (4.2%, *P*‐value 0.0024) and 43 (27%, *P*‐value 8.40 × 10^−17^) showed DAS in the *rs31‐1* and 35S::RS31 lines, respectively. Additionally, 12 (7.5%, *P*‐value 0.040) of these genes have At‐RS31 binding sites identified by iCLIP, with six of these showing DAS in the At‐RS31 overexpression plants, suggesting that they could be direct targets for TOR‐mediated regulation of alternative splicing via At‐RS31.

Among these putative targets, CIPK3, a kinase regulating ABA stress responses and seed development (Kim *et al*., 2003), has an At‐RS31 binding site 20 nt upstream of an unannotated CE containing a PTC (Table S8; Fig. 5b). At‐RS31 promotes exclusion of this exon, potentially increasing the proportion of the full‐length protein. Notably, CIPK3 interacts with RAPTOR1B and may negatively regulate TORC activity by phosphorylation (Li *et al*., 2023), suggesting a link between At‐RS31‐mediated splicing and TOR activity, particularly under ABA‐dependent conditions.

Further analysis of At‐RS31 iCLIP targets and DAS genes (Tables S4, S8) revealed additional TOR targets not present in the initial data set (Riegler *et al*., 2021). The *MCM3* gene (AT5G46280), DNA replication helicase and a known TOR marker gene (Yamamoto *et al*., 2018; Jamsheer *et al*., 2022b), is negatively regulated by At‐RS31. In the *rs31‐1* mutant, *MCM3* intron retention decreases, while it increases in 35S::RS31 plants (Table S8; Fig. S12), implying a role for alternative splicing in regulating MCM3 protein levels. At‐RS31 binding sites were identified on transcripts of S6K1 kinase and RPS6B ribosomal protein (Table S4), key components of the TOR pathway and TOR phosphorylation targets that modulate the translational capacity of the cell (Dobrenel *et al*., 2016). This raises the possibility that At‐RS31 influences alternative splicing of TOR phosphoproteins, adding a regulatory layer upstream of TOR.

To investigate this further, we examined 160 genes encoding TOR‐phosphorylated proteins (Van Leene *et al*., 2019; Scarpin *et al*., 2020; Fig. 5c; Tables S11, S12). At‐RS31 binding sites were found on the transcripts of 18 of these genes (*P*‐value 1.56 × 10^−4^), and 16 exhibited DAS (*P*‐value 0.0373). Five genes (*ATG13, NIG, P2Y/RPP2B, At‐RS40*, and *At‐RS41*) showed both At‐RS31 binding and DAS, likely representing direct targets of At‐RS31. The shared genes are involved in essential processes, such as metabolism, autophagy, cytoskeleton organization, and RNA splicing (Table S11).

We also investigated proteins interacting with TORC1 components that represent genuine TOR targets and upstream regulators (Jamsheer *et al*., 2022a). Among 167 TORC1 interactors, At‐RS31 binding sites were identified in 13 genes (*P*‐value 0.025), including the TOR target S6K1, while 19 showed DAS (*P*‐value 0.007; Fig. 5d; Tables S11, S12). Several in‐frame DAS events have the potential to generate protein isoforms (Table S11). They include EIs in pyruvate dehydrogenase EMB3003, tetratricopeptide repeat‐like protein TPR1, and eukaryotic translational initiation factor eIF2B‐ε3. In the deubiquitinase UBP12, DAS leads to an isoform differing by one amino acid due to an alternative 3′ splice site. In TTI1, part of the Triple T complex, At‐RS31 modulates an alternative 5′ splice site, leading to the inclusion of six amino acids, possibly influencing TOR activation in response to the glucose/energy status of the cell (Liu & Xiong, 2022). In some genes, DAS occurs in UTRs, whose impact is not always clear (Table S11). However, At‐RS31 may modulate the presence of uORFs, as observed for the ABA‐activated SnRK2.8 kinase (Wu & Hsu, 2023), where At‐RS31 decreases intron retention in the 5′ UTR, potentially removing a uORF that may enhance the translation of the main ORF. Multiple At‐RS31 iCLIP crosslinks in the SnRK2.8 5′ UTR suggest a direct regulatory role (Tables S8, S11; Fig. 5e). Given that SnRK2.8 interacts with RAPTOR1B, whose phosphorylation by SnRK2s disrupts the TOR complex to limit growth under stress (Wang *et al*., 2018), At‐RS31 may act upstream, controlling ABA‐dependent TOR inactivation. Additionally, an At‐RS31 binding site is present in the ABA receptor PYL4, which also interacts with TOR (Table S11; Wang *et al*., 2018). Together, several examples suggest a broader role for At‐RS31 in coordinating responses between TOR‐mediated growth control and ABA signalling.

The overlap of genes affected by both TOR modulation and At‐RS31 activity reveals a complex, multi‐level regulatory control. These genes appear to be controlled post‐transcriptionally via At‐RS31‐mediated splicing and possibly at the translational level via TOR‐dependent mechanisms.

### Role of At‐RS31 in integrating abscisic acid metabolism and signalling with the TOR pathway

**Fig. 6:**
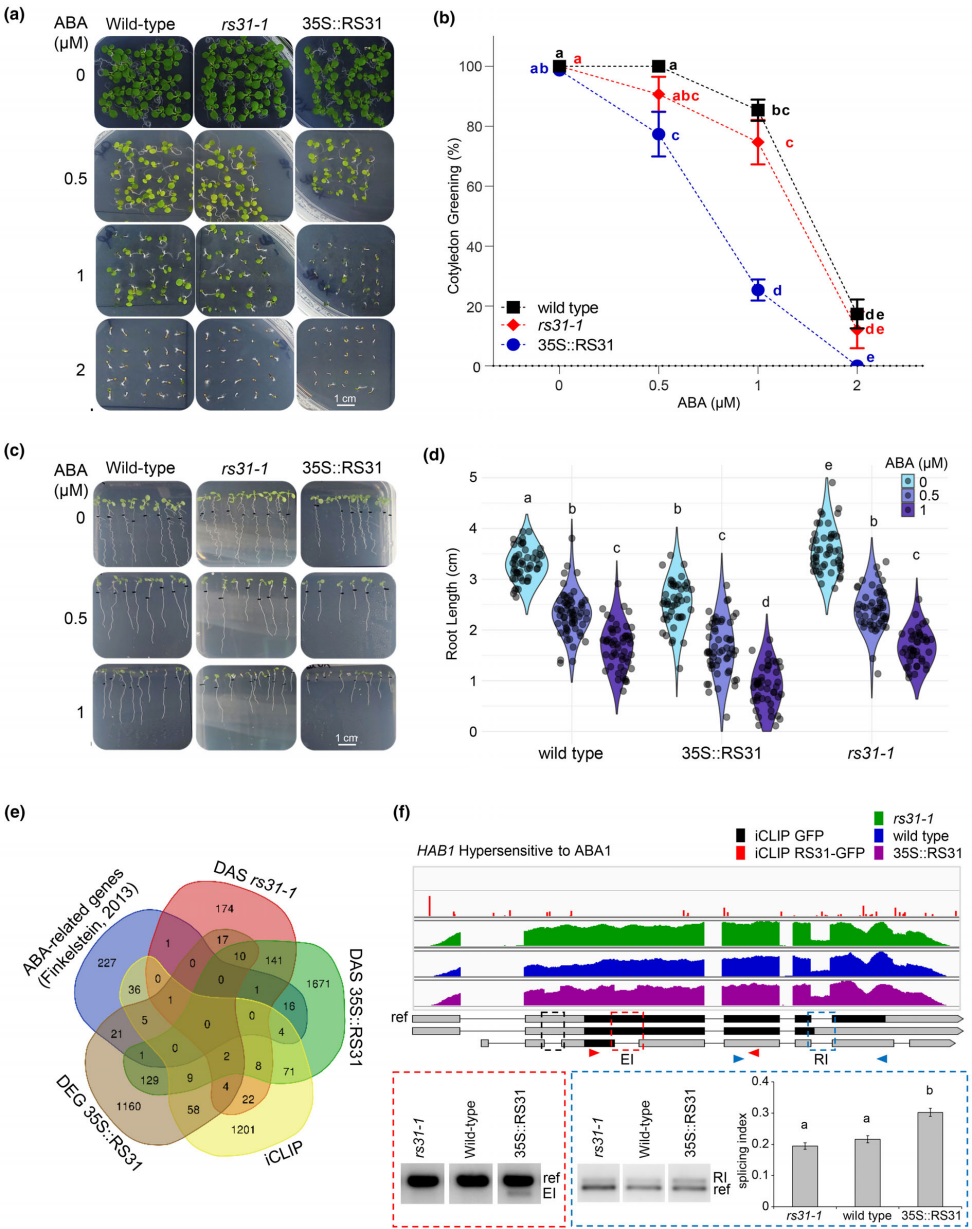
*Arabidopsis thaliana* serine/arginine‐rich (SR) protein At‐RS31 is involved in regulating abscisic acid (ABA) pathway. (a–d) Phenotypic analysis of *rs31‐1*, 35S::RS31 and wild‐type (WT) seedlings in response to different ABA concentrations. (a, c) Representative images of cotyledon greening (a) and root growth (c). (b) Percentage of seedlings with green and open cotyledons after 9 d. Data represent mean ± SE of three independent biological replicates (*n* = 25 seedlings per genotype per replicate). Different letters indicate statistically significant differences (*P* < 0.05 by ANOVA of transformed distribution followed by Tukey's *post hoc* test). (d) Root length measurements after 9 d. Violin plots show the range of the distribution of data across six biological replicates (*n* = 8–15 seedlings per replicate). The median is represented by the widest part of each plot. Individual points correspond to the lengths of individual roots. Different letters indicate statistically significant differences (ANOVA followed by Tukey's *post hoc* test). (e) Venn diagram comparison between genes undergoing differential alternative splicing (DAS) or differential expression (DE) in *rs31‐1* or 35S::RS31, genes with At‐RS31 binding sites identified by individual‐nucleotide resolution crosslinking and immunoprecipitation (iCLIP), and ABA‐related genes. (f) At‐RS31 modulates alternative splicing of *HAB1* (*HYPERSENSITIVE TO ABA1*). Integrated Genomics Viewer tracks show iCLIP crosslink sites for green fluorescent protein (GFP) and RS31‐GFP, alongside RNA sequencing read coverage for *rs31‐1*, 35S::RS31, and WT plants. The related tracks have the same scale. In the transcript models, protein‐coding regions (from the translational start codon to the stop codon or premature termination codon) are shown in black. Only transcripts relevant to the DAS events are shown, with dashed rectangles marking these alternatively spliced regions. Representative reverse transcriptase polymerase chain reaction (RT‐PCR) gels show *HAB1* isoforms in the three genotypes. Colour‐coded arrowheads indicate primers. EI, exitron; ref, reference transcript; RI, intron retention. Bar graphs show splicing index values (mean ± SE, *n* = 3). Different letters indicate statistically significant differences (*P* < 0.05, ANOVA followed by Fisher's LSD test). RS, arginine/serine.

We observed that among genes that undergo DAS upon TOR inhibition or encode TOR‐dependent phosphoproteins and TORC1 interactors (Table S11), At‐RS31 binds and modulates the alternative splicing of the transcripts of several important genes related to the ABA pathway. Moreover, functional enrichment analysis of At‐RS31 targets revealed GO terms associated with response to ABA and other stresses (Table S5). Increased ABA levels inhibit TOR signalling, thereby restricting growth under stress, while TOR counteracts ABA signalling to promote growth under favourable conditions. This interplay balances plant growth and stress responses (Wang *et al*., 2018). Given that At‐RS31 is regulated by TOR (Riegler *et al*., 2021) and influences alternative splicing of TOR‐related genes (Table S11), including those involved in the ABA pathway, we hypothesized that At‐RS31 serves as a molecular link between the TOR and ABA pathways. To test this hypothesis, we examined the effects of At‐RS31 overexpression and knockout on ABA responses in Arabidopsis seedlings. While *rs31‐1* seedlings exhibited responses similar to WT plants, At‐RS31 overexpression significantly enhanced ABA sensitivity, resulting in marked growth inhibition, paler appearance, and defects in cotyledon greening (Fig. 6a–d). These phenotypes, including impaired root growth and cotyledon greening, were more pronounced upon ABA treatment in the 35S::RS31 line (Fig. 6a–d), suggesting a role for At‐RS31 in amplifying ABA‐mediated growth inhibition.

To determine whether these phenotypes are linked to the regulation of ABA‐related genes by At‐RS31, we analysed its potential influence on a compiled set of 313 genes (Finkelstein, 2013), covering genes involved in ABA metabolism, transport, signalling, and core ABA‐induced and ABA‐repressed genes. Out of these 313 genes, 86 are linked to At‐RS31 based on DAS, DE changes, or the presence of At‐RS31 binding sites identified by iCLIP (*P*‐value 5.52 × 10^−10^; Fig. 6e; Tables S12, S13). Within this group of 86 genes, 46 had the binding sites (*P*‐value 1.96 × 10^−13^), including genes involved in ABA synthesis (*NCED6*), catabolism (*CYP707A3*), conjugation (*UGT73B1*), and transport (*ABCG25*). At‐RS31 also binds transcripts of the ABA receptors PYL4 and PYL5, as well as GUN5/ABAR/CHLH, which plays a role in chloroplast retrograde signalling and antagonizes the WRKY18/40/60 transcription repressors of ABA signalling. Additionally, transcripts of several key TFs, phosphatases, and kinases involved in ABA metabolism and signalling (e.g. WRKY18, WRKY40, AHG3, FRY2/CPL1, CRK36, CIPK3, and CPK32) were identified as At‐RS31 targets. Notably, At‐RS31 binding sites were more prevalent in core ABA‐repressed genes (17 out of 47) than in ABA‐induced genes (2 out of 68; Finkelstein, 2013; Table S13). Moreover, At‐RS31 upregulates core ABA‐repressed genes while downregulating ABA‐induced genes. In total, 28 ABA‐related genes were present among 35S::RS31 DEGs (*P*‐value 1.66 × 10^−4^; Tables S9, S12, S13).

At‐RS31 also influences alternative splicing in ABA‐related genes, although the number of affected genes is relatively low (three genes in *rs31‐1* and 22 in 35S::RS31; Table S13). Nevertheless, even this limited number of modulated splicing events could have meaningful impacts on the ABA pathway. The affected genes span various functional categories critical to ABA signalling and function, including ABA synthesis (*ABA3*), transport (*ABCG40/PDR12*), transcription regulation (e.g. *EEL, HAT1*, and *ABF3*), and ABA signalling components (Tables S8, S13).

Beyond DAS events in *CIPK3* and *SnRK2.8* (Fig. 5b,e) described above, HAB1 represents another significant example of an ABA signalling component influenced by At‐RS31. HAB1, a PP2C phosphatase, is a negative regulator of ABA signalling that dephosphorylates and inactivates SnRK2 kinases (Wang *et al*., 2018). We detected three DAS events in *HAB1* (Table S8; Fig. 6f). At‐RS31 promotes the removal of an intron within the 5′ UTR, and also the removal of an EI in the first coding exon, introducing a PTC. These novel DAS events reduce the *HAB1.1* splice variant, which encodes the full‐length protein. Importantly, At‐RS31 also promotes retention of an intron between the third and fourth coding exons, generating the *HAB1.2* isoform, known to upregulate ABA signalling by acting antagonistically to *HAB1.1* (Wang *et al*., 2015).

Taken together, the regulation of *SnRK2.8* and *CIPK3* by At‐RS31, both of which interact with the TOR complex and may inhibit its activity, is consistent with the need for enhanced ABA signalling under stress conditions. Additionally, modulation of the *HAB1* splicing by At‐RS31 to favour the *HAB1.2* isoform, which enhances ABA signalling, further supports its role in balancing ABA and TOR pathways. At‐RS31 modulates the expression of a wide range of ABA‐related genes. By promoting alternative splicing events that enhance or repress specific ABA pathway components, At‐RS31 provides an additional regulatory layer that integrates ABA and TOR pathways. This integration ensures that under stress conditions, ABA signalling is augmented to inhibit the TOR pathway, prioritizing stress responses over growth. Conversely, under favourable conditions, TOR signalling can regulate At‐RS31 to support growth by modulating ABA signalling components. Consistently, the overexpression line of *At‐RS31* has an ABA hypersensitive phenotype (Fig. 6a–d). These data show that At‐RS31 helps balance growth and stress responses.

### Conclusion

Using *in vivo* iCLIP and *in vitro* RNAcompete, we identified consistent RNA‐binding motifs for At‐RS31, such as CAGA‐containing sequences. While both methods revealed overlapping binding preferences, iCLIP uncovered additional motifs, including CU‐rich sequences enriched upstream of 5′ splice sites. This suggests that the RNA‐binding specificity of At‐RS31 *in planta* is influenced in a context‐dependent manner, for example, by post‐translational modifications and protein–protein interactions, underscoring the relevance of combining *in vivo* and *in vitro* approaches to fully characterize properties of RBPs.

At‐RS31 modulates alternative splicing of key genes within the TOR pathway, linking it to the regulation of growth and stress responses. As At‐RS31 itself is regulated by TOR, it acts as a molecular integrator between TOR and ABA signalling. Under stress, At‐RS31 enhances ABA signalling, inhibiting TOR‐mediated growth to prioritize stress adaptation. Conversely, under favourable conditions, TOR signalling modulates *At‐RS31* to balance ABA responses and optimize growth.

Finally, alternative splicing is a critical mechanism regulating At‐RS31 and other plant SR proteins. In particular, we show that extensive cross‐regulation of alternative splicing among different SR proteins likely contributes to maintaining stability within the splicing regulatory network. In this sense, a positive feedback loop between At‐RS31 and At‐SR30 amplifies their protein‐coding isoforms in response to environmental cues, such as light and sugars, mediated by chloroplast retrograde signalling and TOR activity. Together, our findings position At‐RS31 as an important regulator of alternative splicing, broadly impacting plant adaptation by integrating stress responses, photosynthesis, and growth through coordinated regulation of the TOR and ABA signalling pathways.

## Competing interests

None declared.

## Author contributions

MK, AB and DS conceived the study. MK, AB, DS, TH and QM acquired the funding. AF prepared the iCLIP and RNAcompete constructs, generated plants for iCLIP, and analysed protein localization. TK performed the iCLIP experiments. ML performed the bioinformatics analyses of the iCLIP data. HZ expressed proteins for the RNAcompete assay, and DR conducted the assay. YM prepared the RNA‐seq libraries. PV and YM conducted the bioinformatics analyses of the RNA‐seq data. BAN, SF, EP, FSR, FEA, RST and SR carried out the RT‐PCR experiments. FSR, FEA, RST and EP conducted the phenotype analyses. MK, DS and EP critically analysed the data and wrote the manuscript. All authors approved the final manuscript. TK, PV and ML contributed equally to this work.

## Disclaimer

The New Phytologist Foundation remains neutral with regard to jurisdictional claims in maps and in any institutional affiliations.

## Supporting information


**Fig. S1** *Arabidopsis thaliana* At‐RS31 protein domain structure, transcript models, and mutant and overexpression lines used in the RNA sequencing.
**Fig. S2** At‐RS31‐GFP fusion protein expressed in transgenic plants used in the individual‐nucleotide resolution crosslinking and immunoprecipitation.
**Fig. S3** Glutathione S‐transferase‐tagged At‐RS31 proteins used for RNAcompete.
**Fig. S4** Intracellular localization of At‐RS31‐GFP fusion protein.
**Fig. S5** Expression of At‐RS31‐GFP fusion protein and its effect on alternative splicing of *At‐RS31* paralog *At‐RS31a* in the *rs31‐1* mutant background.
**Fig. S6** Immunopurification of At‐RS31 protein–RNA complexes from ultraviolet crosslinked RS31::RS31‐GFP and 35S::GFP plants and preparation of individual‐nucleotide resolution crosslinking and immunoprecipitation libraries.
**Fig. S7** Genome‐wide distribution of crosslink sites.
**Fig. S8** Sequence logo of At‐RS31 binding sites enriched upstream of 5′ splice sites.
**Fig. S9** RNAcompete analysis of the *Arabidopsis thaliana* At‐RS31 protein.
**Fig. S10** Reverse transcription polymerase chain reaction analyses of differential alternative splicing events in genes with At‐RS31 binding sites identified by individual‐nucleotide resolution crosslinking and immunoprecipitation.
**Fig. S11** Reverse transcription polymerase chain reaction analyses of differential alternative splicing in genes encoding RNA‐binding proteins and splicing factors, including Serine/Arginine‐rich proteins.
**Fig. S12** Example of At‐RS31 and Target of Rapamycin pathway shared target MCM3 MINICHROMOSOME MAINTENANCE 3 (AT5G46280).

**Table S1** Oligonucleotides used in this study.
**Table S2** Individual‐nucleotide resolution crosslinking and immunoprecipitation read statistics.
**Table S3** Coordinates of At‐RS31 binding sites identified by individual‐nucleotide resolution crosslinking and immunoprecipitation in *Arabidopsis thaliana*.
**Table S4** At‐RS31 target transcripts identified by individual‐nucleotide resolution crosslinking and immunoprecipitation in *Arabidopsis thaliana*.
**Table S5** Functional enrichment analysis.
**Table S6** Distances from transcription start sites to At‐RS31 binding sites in *Arabidopsis thaliana*.
**Table S7** Regions upstream of 5′ splice sites containing At‐RS31 binding sites in *Arabidopsis thaliana*.
**Table S8** Differential alternative splicing analysis for At‐RS31 mutant and overexpression plants.
**Table S9** Differential gene expression analysis for At‐RS31 mutant and overexpression plants.
**Table S10** Transcription factors modulated by At‐RS31.
**Table S11** Shared targets of At‐RS31 and the Target of Rapamycin pathway in *Arabidopsis thaliana*.
**Table S12** Contingency tables for statistical analysis of gene overlaps.
**Table S13** At‐RS31 in abscisic acid metabolism and signalling in *Arabidopsis thaliana*.
Please note: Wiley is not responsible for the content or functionality of any Supporting Information supplied by the authors. Any queries (other than missing material) should be directed to the *New Phytologist* Central Office.

## Data Availability

RNA‐seq reads have been deposited at the Short Read Archive under the accession nos.: SRX2640702, SRX2640703, SRX2640704, SRX2640706, SRX2640707, SRX2640708, SRX2640710, SRX2640711, and SRX2640712. iCLIP reads have been submitted to SRA under the BioProject accession no.: PRJNA1179310.
