# Supplementary material for: At‐RS31 orchestrates hierarchical cross‐regulation of splicing factors and integrates alternative splicing with TOR‐ABA pathways
Source: New Phytol. 2025 May 26;247(2):738–59. doi: 10.1111/nph.70221 (PMC12177302; doi:10.1111/nph.70221)
Supplement: Supplementary file 1 — Fig. S1 Arabidopsis thaliana At‐RS31 protein domain structure, transcript models, and mutant and overexpression lines used in the RNA sequencing. Fig. S2 At‐RS31‐GFP fusion protein expressed in transgenic plants used in the individual‐nucleotide resolution crosslinking and immunoprecipitation. Fig. S3 Glutathione S‐transferase‐tagged At‐RS31 proteins used for RNAcompete. Fig. S4 Intracellular localization of At‐RS31‐GFP fusion protein. Fig. S5 Expression of At‐RS31‐GFP fusion protein and its effect on alternative splicing of At‐RS31 paralog At‐RS31a in the rs31‐1 mutant background. Fig. S6 Immunopurification of At‐RS31 protein–RNA complexes from ultraviolet crosslinked RS31::RS31‐GFP and 35S::GFP plants and preparation of individual‐nucleotide resolution crosslinking and immunoprecipitation libraries. Fig. S7 Genome‐wide distribution of crosslink sites. Fig. S8 Sequence logo of At‐RS31 binding sites enriched upstream of 5′ splice sites. Fig. S9 RNAcompete analysis of the Arabidopsis thaliana At‐RS31 protein. Fig. S10 Reverse transcription polymerase chain reaction analyses of differential alternative splicing events in genes with At‐RS31 binding sites identified by individual‐nucleotide resolution crosslinking and immunoprecipitation. Fig. S11 Reverse transcription polymerase chain reaction analyses of differential alternative splicing in genes encoding RNA‐binding proteins and splicing factors, including Serine/Arginine‐rich proteins. Fig. S12 Example of At‐RS31 and Target of Rapamycin pathway shared target MCM3 MINICHROMOSOME MAINTENANCE 3 (AT5G46280). [file NPH-247-738-s001.pdf]

SUPPORTING INFORMATION

At-RS31 orchestrates hierarchical cross-regulation of splicing factors and integrates alternative splicing with TOR-ABA pathways

Tino Köster[1,*], Peter Venhuizen[2,*], Martin Lewinski[1,*], Ezequiel Petrillo[3], Yamile Marquez[4], Armin Fuchs[4], Debashish Ray[5], Barbara A. Nimeth[2], Stefan Riegler[2], Sophie Franzmeier[2], Florencia S. Rodríguez[3], Federico E. Aballay[3], Rocío S. Tognacca[3], Hong Zheng[5], Timothy Hughes[5,6], Quaid Morris[7,8], Andrea Barta[4], Dorothee Staiger[1,+], Maria Kalyna[2,+]

[1] RNA Biology and Molecular Physiology, Faculty of Biology, Bielefeld University, 33615 Bielefeld, Germany

[2] Institute of Molecular Plant Biology, Department of Biotechnology and Food Science, BOKU University, 1190 Vienna, Austria.

[3] Instituto de Fisiología, Biología Molecular y Neurociencias (IFIBYNE), 1428 Buenos Aires, Argentina

[4] Max Perutz Labs, Medical University Vienna, Vienna BioCenter (VBC), 1030 Vienna, Austria

[5] Donnelly Centre, University of Toronto, M5S 3E1 Toronto, ON Canada

[6] Department of Molecular Genetics, University of Toronto, M5S 3E1 Toronto, ON Canada

[7] Sloan Kettering Institute, Memorial Sloan Kettering Cancer Center, NY 10065 New York, NY, USA

[8] Graduate Program in Computational Biology and Medicine, Weill-Cornell Graduate School, NY 10065 New York, NY, USA

[+]Authors for correspondence:

Maria Kalyna mariya.kalyna@boku.ac.at

Dorothee Staiger dorothee.staiger@uni-bielefeld.de

* These authors contributed equally to this work

Article acceptance date: 11 April 2025

The following Supporting Information is available for this article:

SUPPLEMENTAL FIGURES

Fig. S1

***Arabidopsis thaliana* At-RS31 protein domain structure, transcript models and mutant and overexpression lines, used in the RNA sequencing**

(a) Schematic of the At-RS31 protein domain structure. The protein contains two RNA recognition motifs (RRM1 and RRM2) and an arginine/serine-rich region (RS).

(b) Structure of At-*RS31* gene, its alternative splicing events and transcript models. Carets indicate alternative splicing events in the second intron. The solid caret represents the splicing event that produces the reference transcript AT3G61860.1 (mRNA1), which encodes the At-RS31 protein. Retention of the second intron generates the mRNA4 transcript. The dashed caret marks the use of an alternative 3' splice site, resulting in the mRNA3 transcript. Dotted carets denote an alternative exon generated via use of both alternative 3' and 5' splice sites, producing the mRNA2 variant. PTC - the premature termination codon. Regions from the AUG start codon to the reference stop codon (mRNA1) or the PTC (mRNA2-4) are shaded in black. The arrowhead indicates the T-DNA insertion site in the SALK_021332 line (*rs31-1* mutant). Black arrows show positions of primers used for reverse transcription polymerase chain reaction (RT-PCR) analysis in (C).

(c) RT-PCR analysis of At-*RS31* transcript levels in the *Arabidopsis thaliana* wild type, 35S::RS31 (overexpression of the mRNA1 under the CaMV 35S promoter), and *rs31-1* plants. Ubiquitin (*UBQ*) was used as a loading control.

(d) Phenotypes of wild type, 35S::RS31 overexpression, and *rs31-1* mutant plants used for RNA-sequencing. Plants were grown under a 16 hour light/8 hour dark cycle at 22°C on vertical agar plates containing half-strength Murashige and Skoog medium.

Fig. S2

At-RS31-GFP fusion protein expressed in transgenic plants used in the individual-nucleotide resolution crosslinking and immunoprecipitation (iCLIP)

(a) *Arabidopsis thaliana At-RS31* genomic region comprising the coding sequence as well as endogenous promoter, untranslated regions and introns was amplified by polymerase chain reaction (arrows – primers) and cloned upstream of the green fluorescent protein (GFP). AT361860.1 represents the reference gene model of *At-RS31*. Thick line – promoter region; grey and black boxes – exons in the untranslated and coding regions, respectively; thin lines – introns. GFP under the Cauliflower Mosaic Virus (CaMV) 35S promoter was used as a control. The constructs were used to generate RS31::RS31-GFP and 35S::GFP transgenic plant lines used in the iCLIP.

(b) Immunoblotting of the wild type, 35S::GFP and RS31::RS31-GFP plants using anti-GFP antibody. Wild type and 35S::GFP plants were used as negative and positive controls, respectively. Ponceau S staining was performed to check loading. Note that only 1/10th of 35S::GFP sample was loaded.

Fig. S3

Glutathione S-transferase (GST)-tagged At-RS31 proteins used for RNAcompete

(a) N-terminal GST-tagged At-RS31 full length cDNA (*T7::GST-31FL*) and At-RS31 RRM (*T7::GST-31RRMs*) constructs. T7 – T7 promoter; RRM – RNA recognition motif.

(b) Purified GST and GST-tagged At-RS31 full length and RRMs-only fusion proteins were analysed by polyacrylamide gel electrophoresis and subsequent Coomassie staining. Marker positions are indicated.

Fig. S4

Intracellular localisation of At-RS31-GFP fusion protein

Confocal laser scanning microscopy of mesophyll protoplasts of *Arabidopsis thaliana* wild-type, 35S::GFP and genomic At-RS31-GFP plants under control of either their endogenous (*RS31::RS31-GFP*) or CaMV 35S (*35S::RS31-GFP)* promoter. Wild-type and 35S::GFP plants were used as negative and positive controls, respectively. Scale bar represents 10 μm. DIC, differential interference contrast. Auto, chloroplast autofluorescence. GFP, green fluorescent protein.

Fig. S5

Expression of At-RS31-GFP fusion protein and its effect on alternative splicing of *At-RS31* paralog *At-RS31a* in the *rs31-1* mutant background

(a) Reverse transcriptase polymerase chain reaction (RT-PCR) analysis confirming the expression of *RS31::RS31-GFP* in the *rs31-1* mutant background.

(b) RT-PCR analysis of *At-RS31* expression using primers that amplify both the endogenous *At-RS31* and the *RS31::RS31-GFP* transgene. The splicing pattern and expression level of *RS31::RS31-GFP* in the *rs31-1* mutant background closely resemble those of the endogenous *At-RS31* in *Arabidopsis thaliana* wild type plants.

(c) RT-PCR analysis of *At-RS31* paralog *At-RS31a*. In the *rs31-1* mutant background, the alternative splicing pattern of *At-RS31a* is shifted toward the reference isoform encoding the full-length protein. Complementation with *RS31::RS31-GFP* restores the splicing pattern of *At-RS31a* to the state closely resembling that of *A. thaliana* wild type plants.

Transcript models are visualized using the Boxify tool (https://boxify.boku.ac.at/). The regions spanning from the translational start codon to either the stop codon or premature termination codon are depicted in black. The arrowhead indicates the T-DNA insertion site in the *rs31-1* mutant (SALK_021332 line). Black arrows represent the positions of primers used in the RT-PCRs. Alternative splicing isoforms (AS) and reference transcripts (ref) encoding full-length proteins are indicated to the right of the gel images. Irrelevant PCR samples are masked with white rectangles.

Fig. S6

Immunopurification of At-RS31 protein–RNA complexes from ultraviolet crosslinked RS31::RS31-GFP and 35S::GFP plants and preparation of individual-nucleotide resolution crosslinking and immunoprecipitation (iCLIP) libraries

(a, b) Autoradiograms of RS31-GFP (A) and GFP protein-RNA complexes (B) used for library preparation. The regions above the RS31-GFP and GFP proteins containing the co-precipitated RNAs, which were cut out for library preparation, are indicated by the red boxes. The positions of the markers are indicated. GFP – green fluorescent protein.

(c, d) Preparation of iCLIP libraries by polymerase chain reaction (PCR) amplification. The numbers of PCR cycles are indicated. The lengths of the cDNAs correspond to the size of the PCR

product minus the length of the P3/P5Solexa primers and the barcode (128 nucleotides (nt) in total). For each replicate, the low (L), medium (M) and high (H) molecular weight PCRs were combined according to their relative concentrations.

Fig. S7

Genome-wide distribution of crosslink sites

Localization of the crosslink sites of RS31-GFP (red) and GFP alone (green) throughout the *Arabidopsis thaliana* chromosomes (Chr) for each biological replicate. The screenshot was created using the Integrative Genomics Viewer (Robinson *et al.*, 2011). GFP – green fluorescent protein.

Fig. S8

Sequence logo of At-RS31 binding sites enriched upstream of 5' splice sites

Sequence logo from aligned RS31-GFP binding sites, which were enriched upstream of 5’ splice sites.

Fig. S9

RNAcompete analysis of the *Arabidopsis thaliana* At-RS31 protein

RNAcompete assay was performed for the full-length At-RS31 protein (a-d) and its truncated version containing RNA recognition motifs (RRMs) only (e-h).

(a) and (e) Top ten 7-mers for At-RS31 and At-RS31RRMs. Corresponding Z-scores for Set A, Set B, and the average of Set A and Set B (All data) are shown.

(b) and (f) Correlation between 7-mers Z-scores from Set A and Set B.

(c) and (g) Alignment of the top ten 7-mers for Set A, Set B, and Set A+B (All data).

(d) and (h) RNA-binding motifs of At-RS31 and At-RS31RRMs. Logos derived from the top ten 7-mers shown in (a) and (e). Sequence logos are generated by WebLogo at https://weblogo.berkeley.edu/.

Fig. S10

Reverse transcription-polymerase chain reaction (RT-PCR) analyses of differential alternative splicing events in genes with At-RS31 binding sites identified by individual-nucleotide resolution crosslinking and immunoprecipitation (iCLIP)

RT-PCR analyses of differential alternative splicing (DAS) events in *rs31-1* mutant, 35S::RS31 overexpression and *Arabidopsis thaliana* wild-type (wt) plants were performed for genes with At-RS31 binding sites identified by iCLIP. Transcript models for the genes visualized using Boxify tool https://boxify.boku.ac.at/. Regions from translational start codon to stop codon or premature termination codon are shown in black. Red arrowheads show positions of At-RS31 binding sites. Blue squares below the transcript models represent primers used in the RT-PCRs, their positions were identified by Boxify. DAS events and reference transcripts (ref) encoding the full-length proteins are indicated to the right of the gels. RI - retained intron; EI – exitron; AA – alternative acceptor / alternative 3' splice site; AD - alternative donor / alternative 5' splice site. *UBQ1* was used as a loading control.

Fig. S11

Reverse transcription-polymerase chain reaction (RT-PCR) analyses of differential alternative splicing in genes encoding RNA binding proteins and splicing factors, including Serine/Arginine-rich (SR) proteins

RT-PCR analysis was conducted to examine differential alternative splicing (DAS) events in *rs31-1* mutant, 35S::RS31 overexpression, and *Arabidopsis thaliana* wild-type (wt) plants, focusing on genes encoding RNA binding proteins and splicing factors, including SR proteins. Transcript models for these genes are visualized using the Boxify tool (https://boxify.boku.ac.at/). Regions, spanning from the translational start codon to the stop codon or premature termination codon, are depicted in black. Blue squares beneath the transcript models represent the positions of primers used in the RT-PCR experiments, as determined by Boxify. Red arrowheads denote the locations of At-RS31 binding sites identified by individual-nucleotide resolution crosslinking and immunoprecipitation (iCLIP). DAS events and reference transcripts (ref) encoding the full-length proteins are indicated to the right of the gel images. AA – alternative acceptor / alternative 3' splice site; AD – alternative donor / alternative 5' splice site; CE – cassette exon / exon skipping; EI – exitron; RI - retained intron. The loading control used was *UBQ1*.

Fig. S12

Example of At-RS31 and Target of Rapamycin (TOR) pathway shared target MCM3 MINICHROMOSOME MAINTENANCE 3 (AT5G46280)

Integrated genome viewer tracks are shown for RNA sequencing read coverage in *rs31-1*, 35S::RS31 and *Arabidopsis thaliana* wild-type plants and for individual-nucleotide resolution crosslinking and immunoprecipitation (iCLIP) crosslinks for RS31-GFP and GFP control. The related tracks have the same scale. Transcript models are visualized using Boxify tool https://boxify.boku.ac.at/. Regions from translational start codon to the reference stop codon or a premature termination codon are shown in black. Dashed rectangle denotes region undergoing differential alternative splicing in 35S::RS31 or *rs31-1* in comparison to wild-type plants. GFP – green fluorescent protein.

SUPPLEMENTARY TABLES

Table S1

Oligonucleotides used in this study

RT-PCR - reverse transcription-polymerase chain reaction; GST - glutathione S-transferase; iCLIP - individual-nucleotide resolution crosslinking and immunoprecipitation; EI – exitron; RI – retained intron

Table S2

Individual-nucleotide resolution crosslinking and immunoprecipitation (iCLIP) read statistics

Table of read, called peak and binding site counts for each sample and processing step. GFP – green fluorescent protein.

Table S3

Coordinates of At-RS31 binding sites identified by individual-nucleotide resolution crosslinking and immunoprecipitation (iCLIP) in *Arabidopsis thaliana*

Table with genomic coordinates of RS31-GFP binding sites with the corresponding PureClip scores. Controls (GFP) are subtracted. Identifiers of associated protein-coding and non-coding

(labeled NC) genes, as well as the position of the binding sites in the transcript regions (5´ UTR, CDS, intron, 3´ UTR) are indicated. Chr - chromosome; 5’ UTR and 3’ UTR - 5’ and 3’ untranslated regions; CDS - coding sequence.

Table S4

At-RS31 target transcripts identified by individual-nucleotide resolution crosslinking and immunoprecipitation (iCLIP) in *Arabidopsis thaliana*

The gene annotation version was taken from Araport11, but only the reference gene models were considered. Controls (GFP) are subtracted. AGI - Arabidopsis Genome Initiative; 5’ UTR and 3’ UTR - 5’ and 3’ untranslated regions; CDS - coding sequence.

Table S5

Functional enrichment analysis

Functional enrichment analysis of *Arabidopsis thaliana* At-RS31 iCLIP targets, differentially alternatively spliced (DAS) and differentially expressed (DEG) genes in the *rs31-1* mutant and *35S::RS31* overexpressing plants was performed using g:Profiler (Kolberg *et al.*, 2023) at https://biit.cs.ut.ee/gprofiler/gost. Version e110_eg57_p18_4b54a898. Term sources include the Gene Ontology (GO) for BP (biological process), MF (molecular function) and CC (cellular component) categories, as well as KEGG (Kyoto Encyclopedia of Genes and Genomes) pathways. Adjusted p-values were calculated using the g:SCS multiple testing correction method implemented in g:Profiler. iCLIP - individual-nucleotide resolution crosslinking and immunoprecipitation.

Table S6

Distances from transcription start sites to At-RS31 binding sites in *Arabidopsis thaliana*

The positional relationship of At-RS31 binding sites to transcription start sites (TSS) was mapped though intersecting the annotated genomic TSS locations (Zhang *et al.*, 2022). The most 5' TSS for each gene was chosen. Columns with the suffix '1' refer to TSS coordinates and annotation, and columns with the suffix '2' refer to the corresponding At-RS31 binding site coordinates and annotation. Chr – chromosome; AGI - Arabidopsis Genome Initiative; BS dist – distance in nucleotides to a binding site.

Table S7

Regions upstream of 5' splice sites containing At-RS31 binding sites in *Arabidopsis thaliana*

Coordinates of RS31-GFP binding sites extended to 21 nucleotides, enriched upstream of 5' splice sites. AGI - Arabidopsis Genome Initiative; 5' UTR and 3' UTR - 5' and 3' untranslated regions; CDS - coding sequence.

Table S8

Differential alternative splicing analysis for At-RS31 mutant and overexpression plants

Differential alternative splicing (DAS) analysis was performed in the *rs31-1* mutant, 35S::RS31 overexpressing plants compared to *Arabidopsis thaliana* wild-type (WT) controls using RNA-seq data. DAS events were obtained and quantified using Whippet (Sterne-Weiler *et al.*, 2018), which calculates the probability of differential splicing based on a Bayesian framework. DAS events with a probability ≥ 0.9 and an absolute delta percent-spliced-in ($|\Delta PSI|$) ≥ 0.1 were considered to be significant.

The table includes gene expression differences in DAS genes based on the adjusted p-value < 0.05 ($|L2FC| > 1$ not required), with p-values corrected for multiple testing using the Benjamini–Yekutieli method. Classification of DAS genes as RNA-binding proteins or splicing factors (SF-RBP) or transcription factors (TF) was based on lists from (Calixto *et al.*, 2018). At-RS31 iCLIP peak occurrences, their coordinates, and their locations relative genic features are based on data shown in Tables S3 and S4.

AA - alternative acceptor; AD - alternative donor; CE – cassette exon / exon skipping; EI – exitron; RI - retained intron; AGI - Arabidopsis Genome Initiative; TPM – transcripts per million; iCLIP - individual-nucleotide resolution crosslinking and immunoprecipitation; 5utr and 3utr - 5' and 3' untranslated regions; CDS - coding sequence; NA – not applicable.

Table S9

Differential gene expression analysis for At-RS31 mutant and overexpression plants

Differential gene expression analysis was performed in the *rs31-1* mutant, 35S::RS31 overexpressing plants compared to *Arabidopsis thaliana* wild-type (WT) controls using RNA-seq data. A gene was considered significantly differentially expressed (DE) in a contrast group if it had an adjusted p-value < 0.05 and |log2 fold change (L2FC)| ≥ 1. p-values were obtained using a

moderated t-test (Limma R package) and adjusted for multiple testing using the Benjamini–Yekutieli method to control the false discovery rate.

The table includes classification of DE genes as RNA-binding proteins or splicing factors (SF-RBP) or transcription factors (TF), based on lists from (Calixto *et al.*, 2018). At-RS31 iCLIP peak occurrences in DE genes, their coordinates, and their locations relative genic features are based on data shown in Tables S3 and S4.

AGI - Arabidopsis Genome Initiative; TPM – transcripts per million; iCLIP - individual-nucleotide resolution crosslinking and immunoprecipitation; 5utr and 3utr - 5' and 3' untranslated regions; CDS - coding sequence; NA – not applicable.

Table S10

Transcription factors modulated by At-RS31

The table shows transcription factors (TF) undergoing differential alternative splicing (DAS) and differential expression (DE) in the *rs31-1* mutant, *35S::RS31* overexpressing plants compared to *Arabidopsis thaliana* wild-type controls. TF gene list is based on (Calixto *et al.*, 2018). Details on DAS and DE in TF genes are shown in Tables S8 and S9. At-RS31 binding sites in DAS and DE TF genes are based on iCLIP data shown in Tables S3 and S4. AGI - Arabidopsis Genome Initiative; iCLIP - individual-nucleotide resolution crosslinking and immunoprecipitation.

Table S11

Shared targets of At-RS31 and the Target of Rapamycin (TOR) pathway in *Arabidopsis thaliana*

The table summarizes At-RS31 iCLIP binding sites and differential alternative splicing (DAS) events in the *rs31-1* mutant and *35S::RS31* overexpressing plants in genes related to the TOR pathway. It includes genes that show DAS under TOR inhibition (TOR DAS) (Riegler *et al.*, 2021), genes encoding proteins regulated by TOR-dependent phosphorylation (TOR phosphoproteins) (Van Leene *et al.*, 2019; Scarpin *et al.*, 2020), and genes encoding proteins interacting with the TOR complex (TOR interactors) (Jamsheer K *et al.*, 2022). AGI - Arabidopsis Genome Initiative; iCLIP - individual-nucleotide resolution crosslinking and immunoprecipitation; ΔPSI - delta percent-spliced-in; AS – alternative splicing; AA – alternative acceptor / alternative 3' splice site; AD – alternative donor / alternative 5' splice site; CE – cassette exon / exon skipping; EI – exitron;

RI - retained intron; CDS – coding sequence; UTR – untranslated region; NMD – nonsense-mediated mRNA decay; PTC – premature termination codon; NA - not applicable.

Table S12

Contingency tables for statistical analysis of gene overlaps

The statistical significance of gene overlaps was assessed using a hypergeometric-based tool available at http://nemates.org/MA/progs/overlap_stats.html. This tool calculates a representation factor, defined as the ratio of observed overlap to expected overlap, and determines a p-value using the hypergeometric (Fisher's exact) test or, for very large sets, a normal approximation. In our analyses, the p-value was obtained from the hypergeometric test comparing each subset to the 33,602 *Arabidopsis thaliana* genes in TAIR10. Only tables for significant overlaps are shown. ABA – abscisic acid; DAS – differential alternative splicing; DE – differential expression; iCLIP - individual-nucleotide resolution crosslinking and immunoprecipitation; TOR – Target of Rapamycin; TORC1 - Target of Rapamycin Complex 1.

Table S13

At-RS31 in abscisic acid (ABA) metabolism and signaling in *Arabidopsis thaliana*

The table summarizes At-RS31 iCLIP binding sites, differential alternative splicing (DAS) events, and differential expression (DE) in the *rs31-1* mutant and 35S::RS31 overexpressing plants. The upper part of the table lists genes involved in metabolism or transport of ABA or signaling in response to ABA, while the lower part lists core ABA-induced or ABA-repressed genes (Finkelstein, 2013). AGI - Arabidopsis Genome Initiative; iCLIP - individual-nucleotide resolution crosslinking and immunoprecipitation.

References

Calixto CPG, Guo W, James AB, Tzioutziou NA, Entizne JC, Panter PE, Knight H, Nimmo HG, Zhang R, Brown JWS. 2018. Rapid and Dynamic Alternative Splicing Impacts the Arabidopsis Cold Response Transcriptome. *Plant Cell* **30**(7): 1424-1444.

Finkelstein R. 2013. Abscisic Acid synthesis and response. *Arabidopsis Book* **11**: e0166.

Jamsheer K M, Awasthi P, Laxmi A. 2022. The social network of target of rapamycin complex 1 in plants. *Journal of Experimental Botany* **73**(20): 7026-7040.

Kolberg L, Raudvere U, Kuzmin I, Adler P, Vilo J, Peterson H. 2023. g:Profiler—interoperable web service for functional enrichment analysis and gene identifier mapping (2023 update). *Nucleic Acids Research* **51**(W1): W207-W212.

Riegler S, Servi L, Scarpin MR, Godoy Herz MA, Kubaczka MG, Venhuizen P, Meyer C, Brunkard JO, Kalyna M, Barta A, et al. 2021. Light regulates alternative splicing outcomes via the TOR kinase pathway. *Cell Rep* **36**(10): 109676.

Robinson JT, Thorvaldsdóttir H, Winckler W, Guttman M, Lander ES, Getz G, Mesirov JP. 2011. Integrative genomics viewer. *Nat Biotechnol* **29**(1): 24-26.

Scarpin MR, Leiboff S, Brunkard JO. 2020. Parallel global profiling of plant TOR dynamics reveals a conserved role for LARP1 in translation. *Elife* **9**: e58795.

Sterne-Weiler T, Weatheritt RJ, Best AJ, Ha KCH, Blencowe BJ. 2018. Efficient and Accurate Quantitative Profiling of Alternative Splicing Patterns of Any Complexity on a Laptop. *Mol Cell* **72**(1): 187-200 e186.

Van Leene J, Han C, Gadeyne A, Eeckhout D, Matthijs C, Cannoot B, De Winne N, Persiau G, Van De Slijke E, Van de Cotte B, et al. 2019. Capturing the phosphorylation and protein interaction landscape of the plant TOR kinase. *Nature Plants* **5**(3): 316-327.

Zhang R, Kuo R, Coulter M, Calixto CPG, Entizne JC, Guo W, Marquez Y, Milne L, Riegler S, Matsui A, et al. 2022. A high-resolution single-molecule sequencing-based Arabidopsis transcriptome using novel methods of Iso-seq analysis. *Genome Biology* **23**(1): 149.

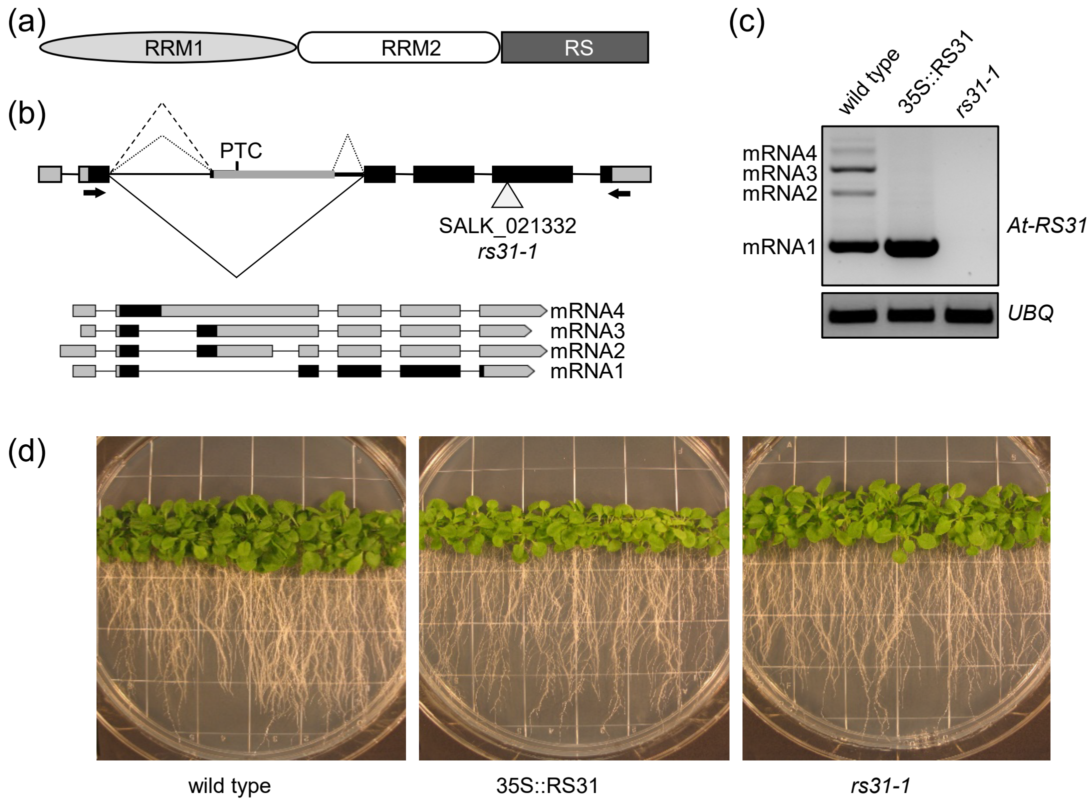

Fig. S1 ***Arabidopsis thaliana*** **At-RS31 protein domain structure, transcript models and mutant and overexpression lines, used in the RNA sequencing**

(a) Schematic of the At-RS31 protein domain structure. The protein contains two RNA recognition motifs (RRM1 and RRM2) and an arginine/serine-rich region (RS).

(b) Structure of At-*RS31* gene, its alternative splicing events and transcript models. Carets indicate alternative splicing events in the second intron. The solid caret represents the splicing event that produces the reference transcript AT3G61860.1 (mRNA1), which encodes the At-RS31 protein. Retention of the second intron generates the mRNA4 transcript. The dashed caret marks the use of an alternative 3' splice site, resulting in the mRNA3 transcript. Dotted carets denote an alternative exon generated via use of both alternative 3' and 5' splice sites, producing the mRNA2 variant. PTC - the premature termination codon. Regions from the AUG start codon to the reference stop codon (mRNA1) or the PTC (mRNA2-4) are shaded in black. The arrowhead indicates the T-DNA insertion site in the SALK_021332 line (*rs31-1* mutant). Black arrows show positions of primers used for reverse transcription polymerase chain reaction (RT-PCR) analysis in (C).

(c) RT-PCR analysis of At-*RS31* transcript levels in the *Arabidopsis thaliana* wild type, 35S::RS31 (overexpression of the mRNA1 under the CaMV 35S promoter), and *rs31-1* plants. Ubiquitin (*UBQ*) was used as a loading control.

(d) Phenotypes of wild type, 35S::RS31 overexpression, and *rs31-1* mutant plants used for RNA-sequencing. Plants were grown under a 16 hour light/8 hour dark cycle at 22°C on vertical agar plates containing half-strength Murashige and Skoog medium.

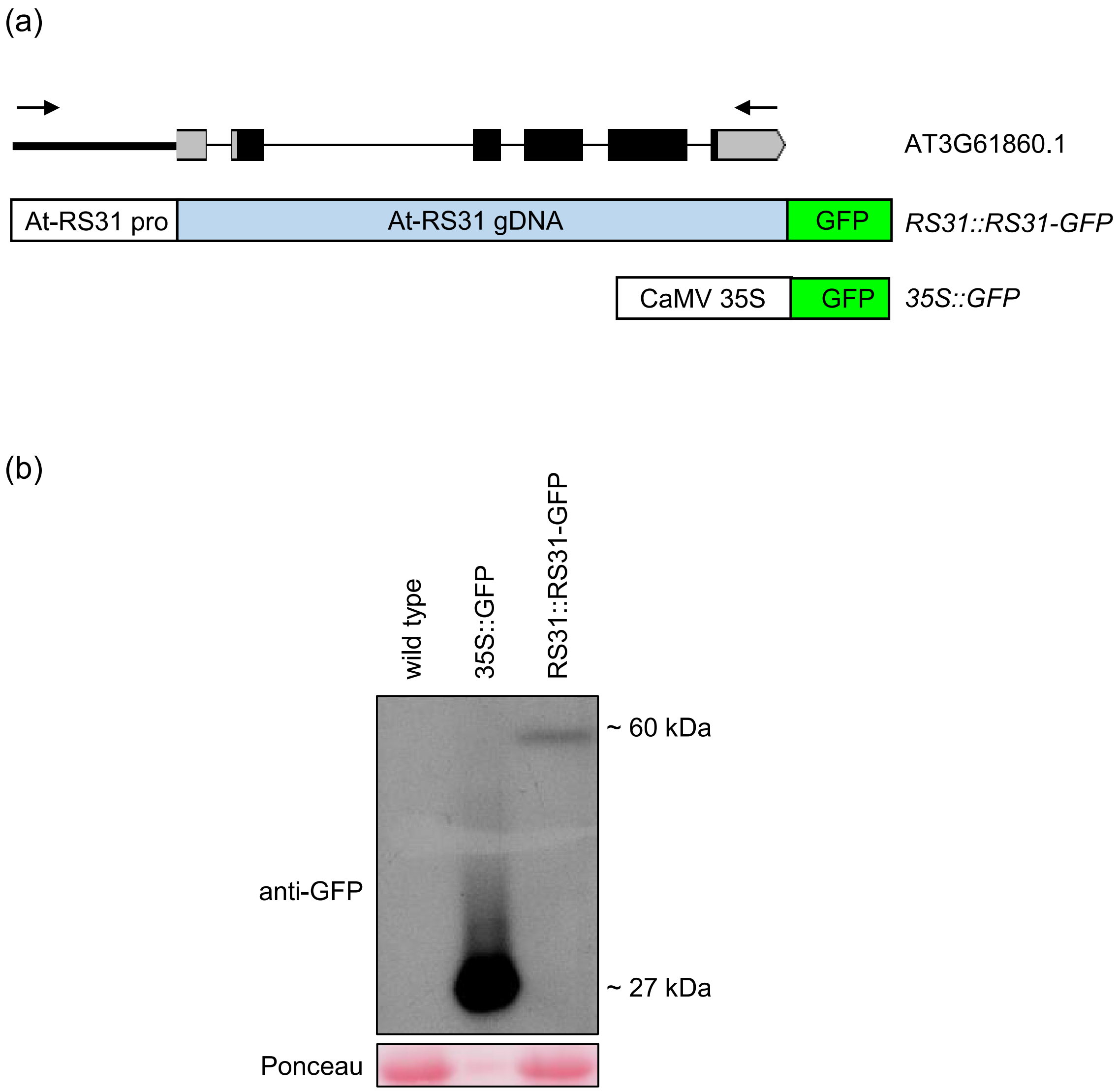

Fig. S2 At-RS31-GFP fusion protein expressed in transgenic plants used in the individual-nucleotide resolution crosslinking and immunoprecipitation (iCLIP)

(a) *Arabidopsis thaliana At-RS31* genomic region comprising the coding sequence as well as endogenous promoter, untranslated regions and introns was amplified by polymerase chain reaction (arrows – primers) and cloned upstream of the green fluorescent protein (GFP). AT361860.1 represents the reference gene model of *At-RS31*. Thick line – promoter region; grey and black boxes – exons in the untranslated and coding regions, respectively; thin lines – introns. GFP under the Cauliflower Mosaic Virus (CaMV) 35S promoter was used as a control. The constructs were used to generate RS31::RS31-GFP and 35S::GFP transgenic plant lines used in the iCLIP.

(b) Immunoblotting of the wild type, 35S::GFP and RS31::RS31-GFP plants using anti-GFP antibody. Wild type and 35S::GFP plants were used as negative and positive controls, respectively. Ponceau S staining was performed to check loading. Note that only 1/10th of 35S::GFP sample was loaded.

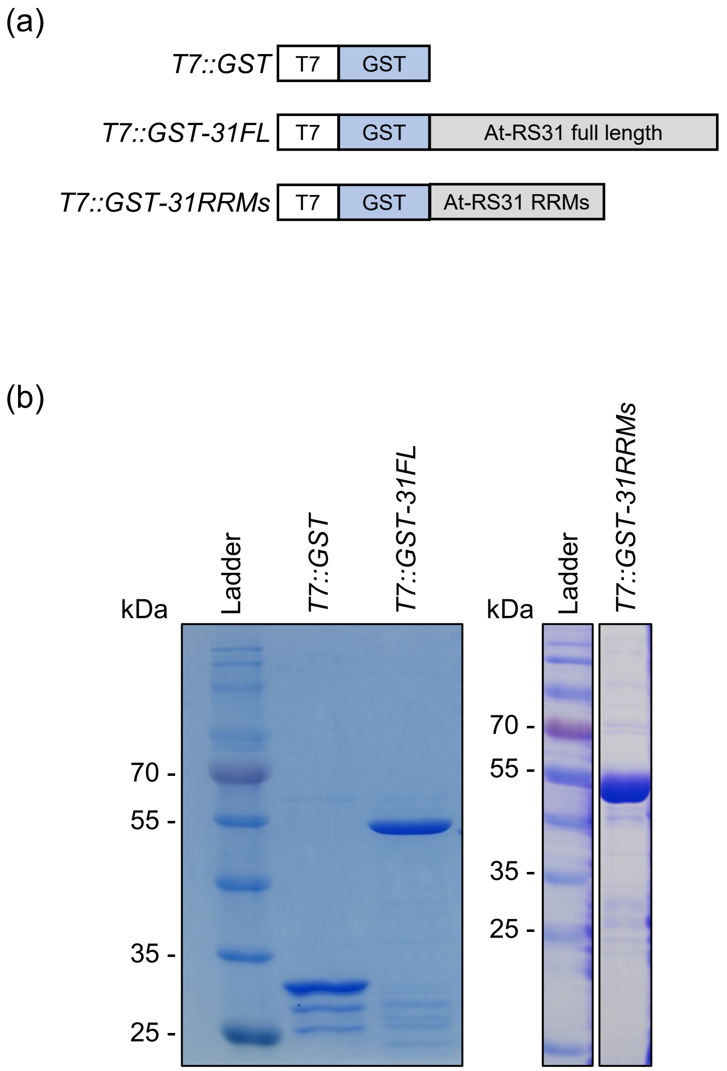

Fig. S3 Glutathione S-transferase (GST)-tagged At-RS31 proteins used for RNAcompete

(a) N-terminal GST-tagged At-RS31 full length cDNA (*T7::GST-31FL*) and At-RS31 RRM (*T7::GST-31RRMs*) constructs. T7 – T7 promoter; RRM – RNA recognition motif.

(b) Purified GST and GST-tagged At-RS31 full length and RRMs-only fusion proteins were analysed by polyacrylamide gel electrophoresis and subsequent Coomassie staining. Marker positions are indicated.

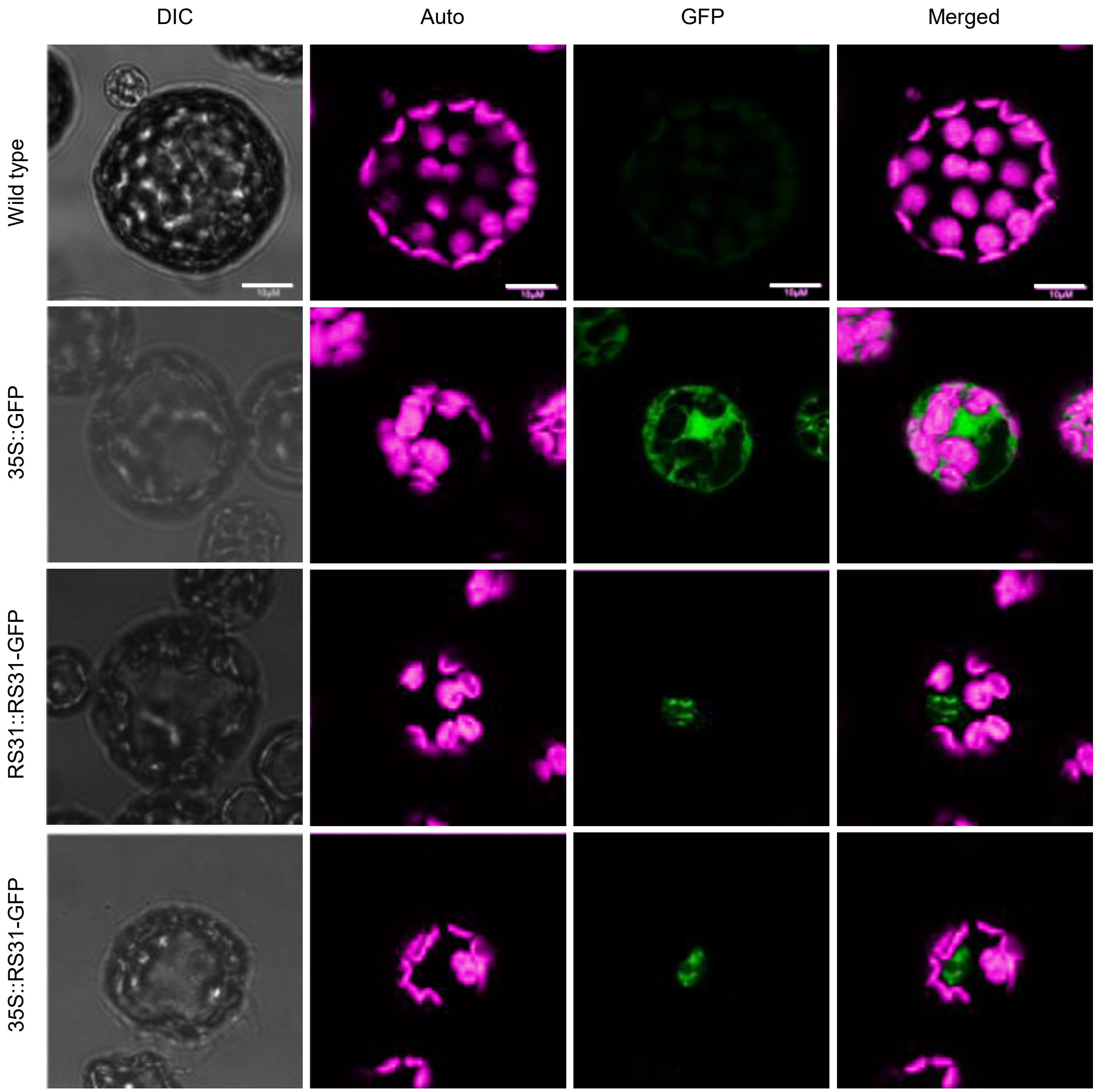

Fig. S4 Intracellular localisation of At-RS31-GFP fusion protein

Confocal laser scanning microscopy of mesophyll protoplasts of *Arabidopsis thaliana* wild-type, 35S::GFP and genomic At-RS31-GFP plants under control of either their endogenous (*RS31::RS31-GFP*) or CaMV 35S (*35S::RS31-GFP)* promoter. Wild-type and 35S::GFP plants were used as negative and positive controls, respectively. Scale bar represents 10 µm. DIC, differential interference contrast. Auto, chloroplast autofluorescence. GFP, green fluorescent protein.

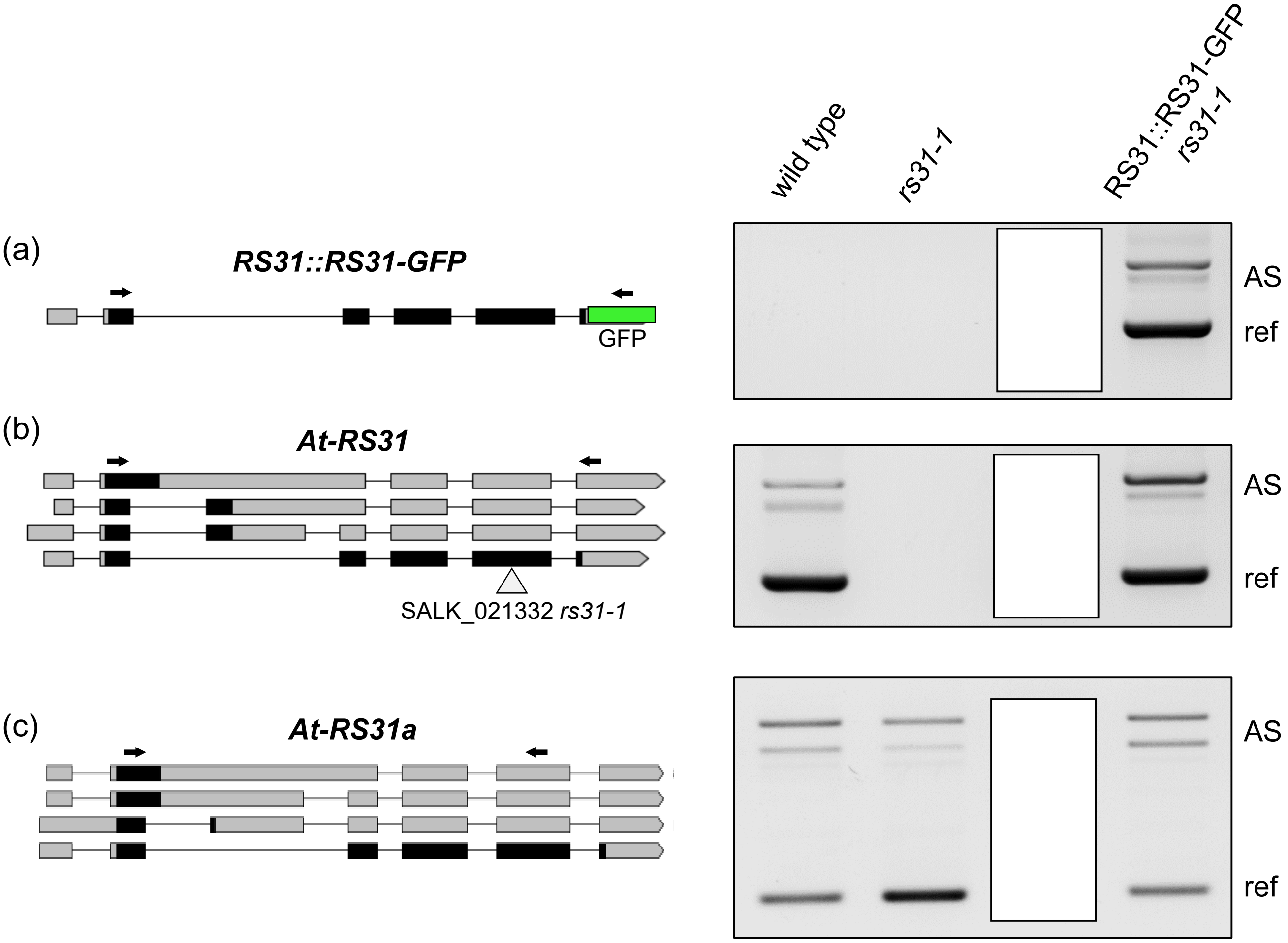

Fig. S5 Expression of At-RS31-GFP fusion protein and its effect on alternative splicing of *At-RS31* paralog *At-RS31a* in the *rs31-1* mutant background

(a) Reverse transcriptase polymerase chain reaction (RT-PCR) analysis confirming the expression of *RS31::RS31-GFP* in the *rs31-1* mutant background.

(b) RT-PCR analysis of *At-RS31* expression using primers that amplify both the endogenous *At-RS31* and the *RS31::RS31-GFP* transgene. The splicing pattern and expression level of *RS31::RS31-GFP* in the *rs31-1* mutant background closely resemble those of the endogenous *At-RS31* in *Arabidopsis thaliana* wild type plants.

(c) RT-PCR analysis of *At-RS31* paralog *At-RS31a*. In the *rs31-1* mutant background, the alternative splicing pattern of *At-RS31a* is shifted toward the reference isoform encoding the full-length protein. Complementation with *RS31::RS31-GFP* restores the splicing pattern of *At-RS31a* to the state closely resembling that of *A. thaliana* wild type plants.

Transcript models are visualized using the Boxify tool (https://boxify.boku.ac.at/). The regions spanning from the translational start codon to either the stop codon or premature termination codon are depicted in black. The arrowhead indicates the T-DNA insertion site in the *rs31-1* mutant (SALK_021332 line). Black arrows represent the positions of primers used in the RT-PCRs. Alternative splicing isoforms (AS) and reference transcripts (ref) encoding full-length proteins are indicated to the right of the gel images. Irrelevant PCR samples are masked with white rectangles.

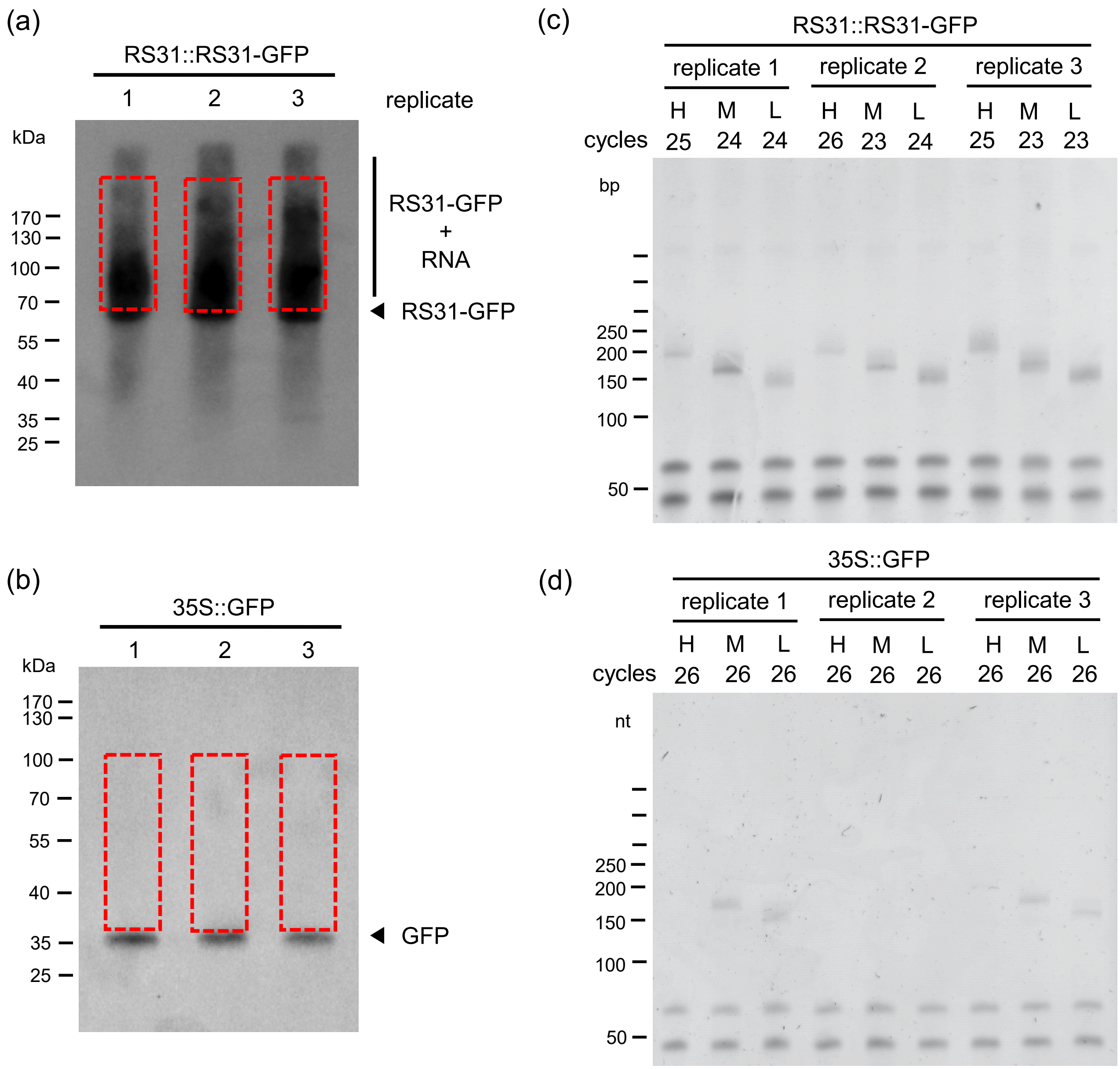

Fig. S6 Immunopurification of At-RS31 protein–RNA complexes from ultraviolet crosslinked RS31::RS31-GFP and 35S::GFP plants and preparation of individual-nucleotide resolution crosslinking and immunoprecipitation (iCLIP) libraries

(a, b) Autoradiograms of RS31-GFP (A) and GFP protein-RNA complexes (B) used for library preparation. The regions above the RS31-GFP and GFP proteins containing the co-precipitated RNAs, which were cut out for library preparation, are indicated by the red boxes. The positions of the markers are indicated. GFP – green fluorescent protein.

(c, d) Preparation of iCLIP libraries by polymerase chain reaction (PCR) amplification. The numbers of PCR cycles are indicated. The lengths of the cDNAs correspond to the size of the PCR product minus the length of the P3/P5Solexa primers and the barcode (128 nucleotides (nt) in total). For each replicate, the low (L), medium (M) and high (H) molecular weight PCRs were combined according to their relative concentrations.

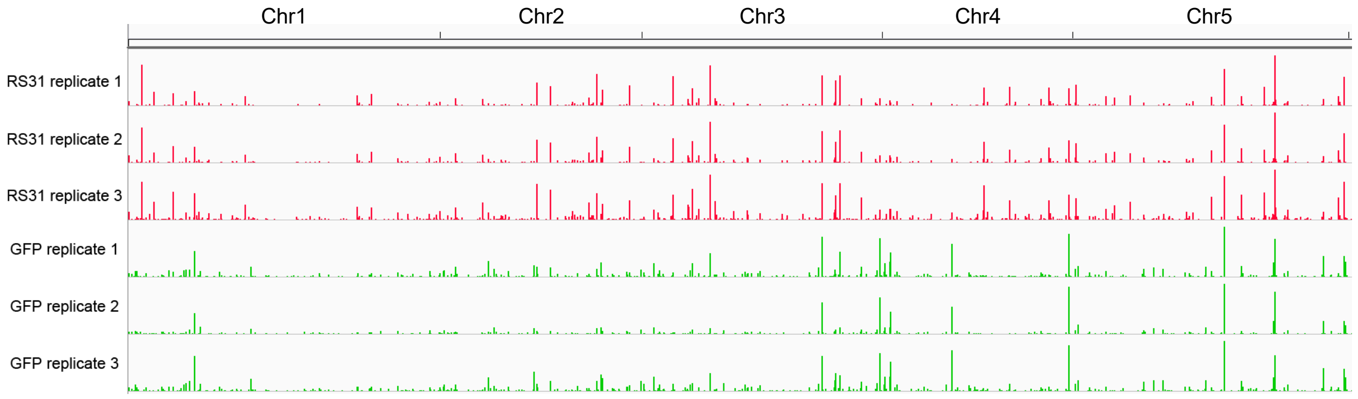

Fig. S7 Genome-wide distribution of crosslink sites

Localization of the crosslink sites of RS31-GFP (red) and GFP alone (green) throughout the *Arabidopsis thaliana* chromosomes (Chr) for each biological replicate. The screenshot was created using the Integrative Genomics Viewer (Robinson et al., 2011). GFP – green fluorescent protein.

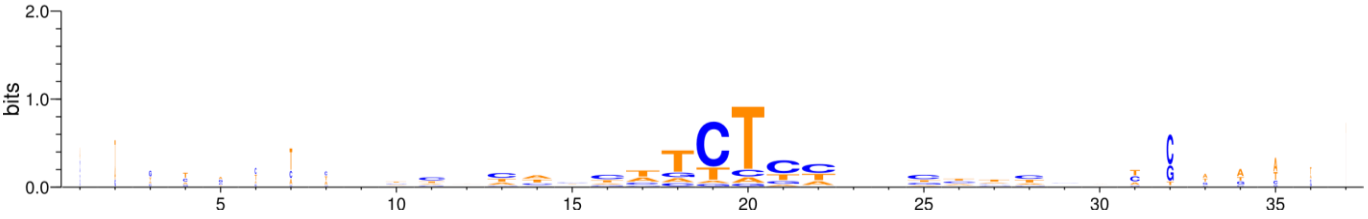

Fig. S8 Sequence logo of At-RS31 binding sites enriched upstream of 5' splice sites

Sequence logo from aligned RS31-GFP binding sites, which were enriched upstream of 5’ splice sites.

At-RS31 full length

(a)

Top 7-mers by Z-score

Set A	score	Set B	score	All data	score
AGACAGA	9.74496159344526	AGACAGA	10.8422841945654	AGACAGA	10.4921902703309
GACAGAA	9.14755284449185	GACAGAA	9.27243790507922	GACAGAA	9.3607258776696
CAGACGA	8.50855234719359	CAGACGA	9.1375585102785	CAGACGA	8.996355988504
ACAGACG	7.96407855067909	ACAGAAG	8.21962929566247	ACAGACG	8.06050069423281
ACAGAGA	7.87333291792667	CAGACAG	8.18965609681787	CAGACAG	7.69229533255235
ACAGACA	7.78258728517426	ACAGACG	7.90491070779412	GACAGAC	7.68078891499983
CAGACAA	7.76746301304886	CAACCAG	7.89741740808296	ACAGAAG	7.6501051348598
AUUCAGA	7.7334334007667	GACAGAC	7.43283282599158	CAGACAA	7.62709229975477
GACAGAC	7.7183091286413	AUCAGAC	7.42159287642486	AUUCAGA	7.48901528912459
AGCAGAC	7.24945669275381	CAGACAA	7.21178048451262	CAGCAGA	7.27039335562682

(b)

7-mer scatter plot

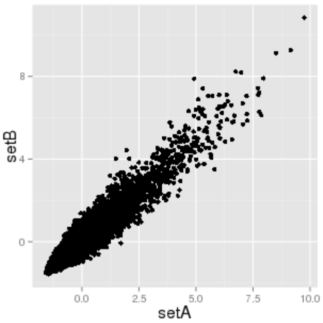

(c)

Aligned top 7-mers

Set A	Set B	All data
---CAGACGA	----CAGACGA	---CAGACGA
---CAGACAA	----CAGACAA	---CAGACAA
AGACAGA---	--GACAGAA--	---CAGACAG
-GACAGAA--	---ACAGAAG-	AGACAGA---
-GACAGAC--	-AGACAGA---	-GACAGAA--
--ACAGAGA-	--GACAGAC--	-GACAGAC--
--ACAGACA-	---ACAGACG-	--ACAGACG-
--ACAGACG-	--AUCAGAC--	--ACAGAAG-
-AGCAGAC--	----CAGACAG	CAGCAGA---
AUUCAGA---	CAACCAG----	AUUCAGA---

(d)

Motif

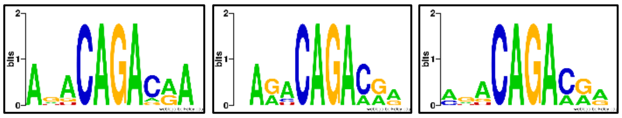

Set A Set B All data

At-RS31 RRMs

(e)

Top 7-mers by Z-score

Set A	score	Set B	score	All data	score
AGACAGA	9.61334606226033	AGACAGA	11.7082288941747	AGACAGA	10.959856529157
AUUCAGA	9.59989449169152	CAACCAG	9.37935910070257	AUUCAGA	9.60672512769926
CAGCAGA	9.29319868272271	AUUCAGA	9.3076607148146	CAGCAGA	9.16483028868364
CAGAACA	8.65021360953369	UUCAGAC	8.89074787835494	GACAGAA	8.85468049484039
GACAGAA	8.64752329541993	CAGACAG	8.83232697133511	UUCAGAC	7.72386885711099
CAGAAGC	8.23590523601441	GACAGAA	8.79249453473069	CAGACGC	7.71837948022881
UCAGACA	8.09600890209881	CAGCAGA	8.65971974604926	CAGACAA	7.70465603802335
CAACAGA	8.06641544684743	AUGCAGA	7.67984180558036	UUCAGAA	7.69916666114117
UUCAGAA	8.02606073514101	CAGACGC	7.63204288165505	CAGAACA	7.61957069634954
ACAGACA	7.95611256818321	CAACCGA	7.61610990701328	CAGAAGC	7.53174066623463

(f)

7-mer scatter plot

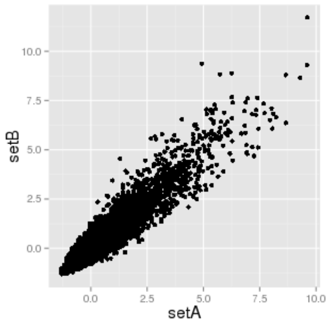

(g)

Aligned top 7-mers

Set A	Set B	All data
AGACAGA---	AGACAGA---	AGACAGA---
-GACAGAA--	-GACAGAA--	-GACAGAA--
--UCAGACA-	---CAGACAG	---CAGACAA
--ACAGACA-	AUUCAGA---	AUUCAGA---
CAGCAGA---	AUGCAGA---	-UUCAGAC--
CAACAGA---	-UUCAGAC--	-UUCAGAA--
AUUCAGA---	---CAGACGC	---CAGAACA
-UUCAGAA--	---CAACCAG	---CAGACGC
---CAGAACA	---CAACCGA	---CAGAAGC
---CAGAAGC	CAGCAGA---	CAGCAGA---

(h)

Motif

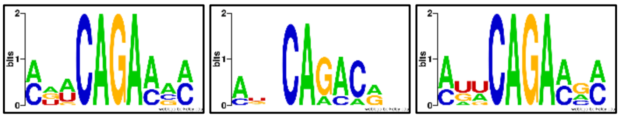

Set A Set B All data

Fig. S9 RNAcompete analysis of the *Arabidopsis thaliana* At-RS31 protein

RNAcompete assay was performed for the full-length At-RS31 protein (a-d) and its truncated version containing RNA recognition motifs (RRMs) only (e-h).

(a) and (e) Top ten 7-mers for At-RS31 and At-RS31RRMs. Corresponding Z-scores for Set A, Set B, and the average of Set A and Set B (All data) are shown.

(b) and (f) Correlation between 7-mers Z-scores from Set A and Set B.

(c) and (g) Alignment of the top ten 7-mers for Set A, Set B, and Set A+B (All data).

(d) and (h) RNA-binding motifs of At-RS31 and At-RS31RRMs. Logos derived from the top ten 7-mers shown in (a) and (e). Sequence logos are generated by WebLogo at https://weblogo.berkeley.edu/.

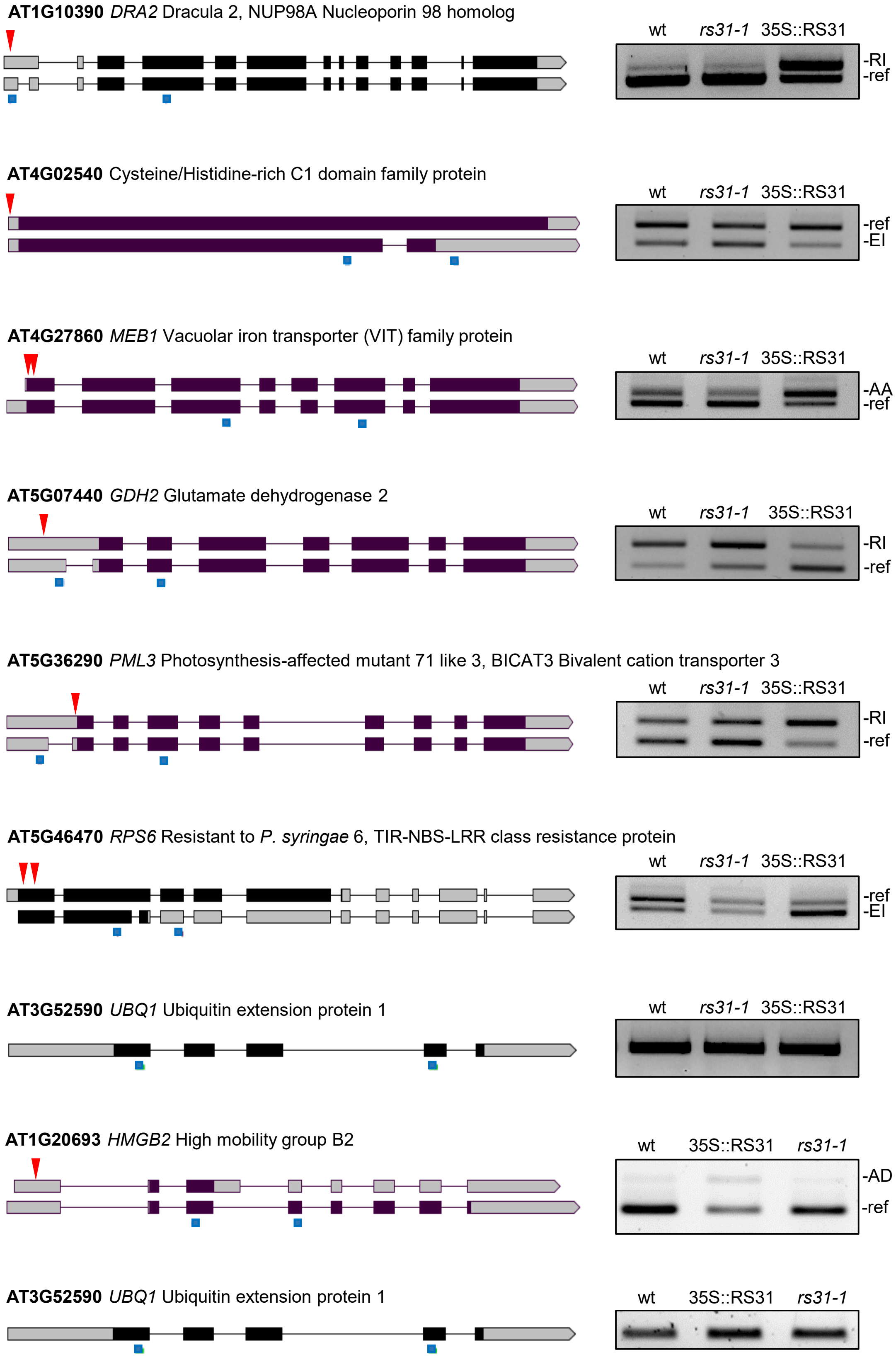

Fig. S10 Reverse transcription-polymerase chain reaction (RT-PCR) analyses of differential alternative splicing events in genes with At-RS31 binding sites identified by individual-nucleotide resolution crosslinking and immunoprecipitation (iCLIP)

Fig.10 (continued): RT-PCR analyses of differential alternative splicing (DAS) events in *rs31-1* mutant, 35S::RS31 overexpression and *Arabidopsis thaliana* wild-type (wt) plants were performed for genes with At-RS31 binding sites identified by iCLIP. Transcript models for the genes visualized using Boxify tool https://boxify.boku.ac.at/. Regions from translational start codon to stop codon or premature termination codon are shown in black. Red arrowheads show positions of At-RS31 binding sites. Blue squares below the transcript models represent primers used in the RT-PCRs, their positions were identified by Boxify. DAS events and reference transcripts (ref) encoding the full-length proteins are indicated to the right of the gels. RI - retained intron; EI – exitron; AA – alternative acceptor / alternative 3' splice site; AD - alternative donor / alternative 5' splice site. *UBQ1* was used as a loading control.

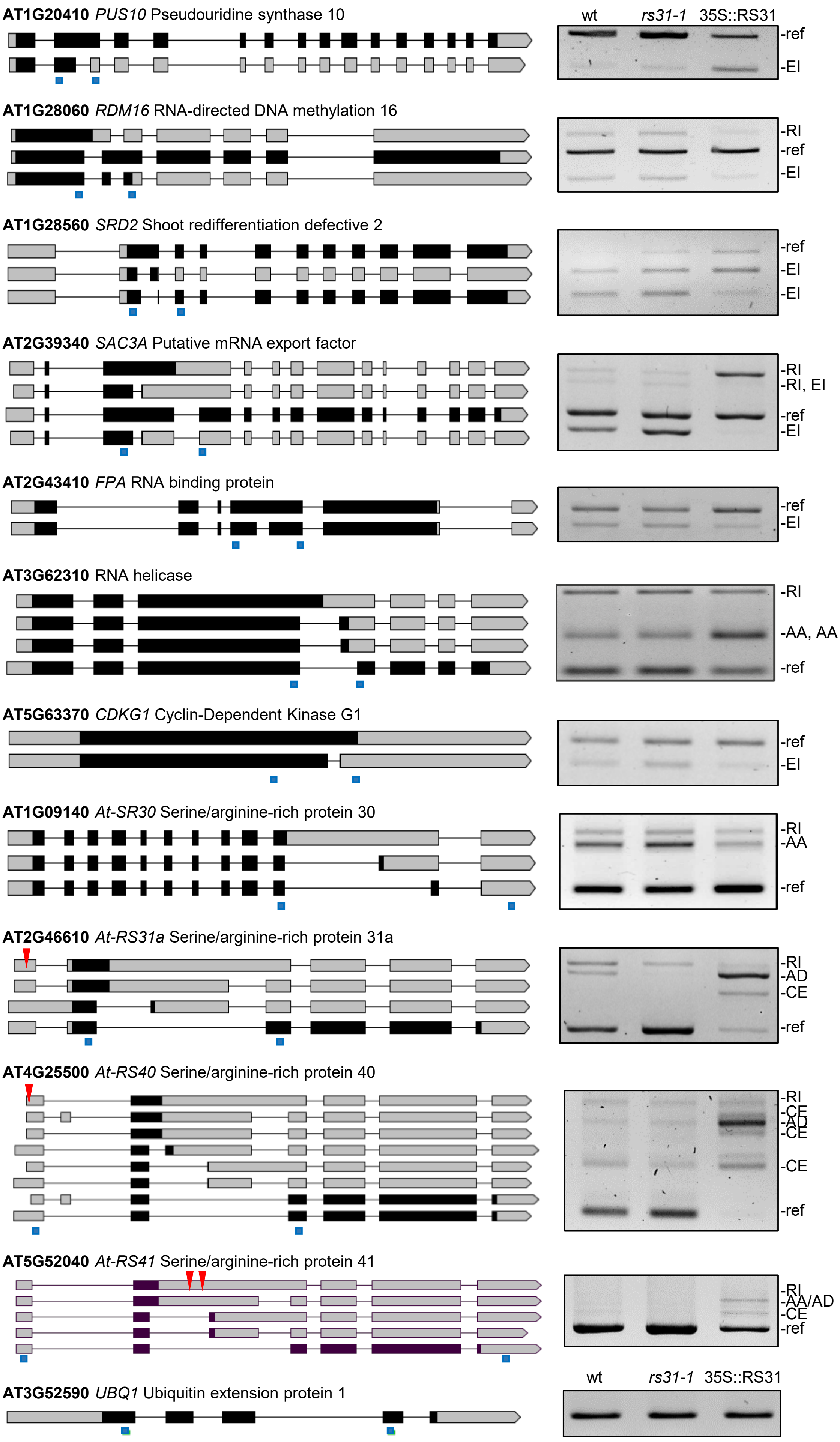
AT1G20410 PUS10 Pseudouridine synthase 10
wt
rs31-1
35S::RS31
-ref
-EI
AT1G28060 RDM16 RNA-directed DNA methylation 16
-RI
-ref
-EI
AT1G28560 SRD2 Shoot redifferentiation defective 2
-ref
-EI
-EI
AT2G39340 SAC3A Putative mRNA export factor
-RI
-RI, EI
-ref
-EI
AT2G43410 FPA RNA binding protein
-ref
-EI
AT3G62310 RNA helicase
-RI
-AA, AA
-ref
AT5G63370 CDKG1 Cyclin-Dependent Kinase G1
-ref
-EI
AT1G09140 At-SR30 Serine/arginine-rich protein 30
-RI
-AA
-ref
AT2G46610 At-RS31a Serine/arginine-rich protein 31a
-RI
-AD
-CE
-ref
AT4G25500 At-RS40 Serine/arginine-rich protein 40
-RI
-CE
-AD
-CE
-CE
-ref
AT5G52040 At-RS41 Serine/arginine-rich protein 41
-RI
-AA/AD
-CE
-ref
AT3G52590 UBQ1 Ubiquitin extension protein 1
wt
rs31-1
35S::RS31

Fig. S11 Reverse transcription-polymerase chain reaction (RT-PCR) analyses of differential alternative splicing in genes encoding RNA binding proteins and splicing factors, including Serine/Arginine-rich (SR) proteins

RT-PCR analysis was conducted to examine differential alternative splicing (DAS) events in *rs31-1* mutant, 35S::RS31 overexpression, and *Arabidopsis thaliana* wild-type (wt) plants, focusing on genes encoding RNA binding proteins and splicing factors, including SR proteins. Transcript models for these genes are visualized using the Boxify tool (https://boxify.boku.ac.at/). Regions, spanning from the translational start codon to the stop codon or premature termination codon, are depicted in black. Blue squares beneath the transcript models represent the positions of primers used in the RT-PCR experiments, as determined by Boxify. Red arrowheads denote the locations of At-RS31 binding sites identified by individual-nucleotide resolution crosslinking and immunoprecipitation (iCLIP). DAS events and reference transcripts (ref) encoding the full-length proteins are indicated to the right of the gel images. AA – alternative acceptor / alternative 3' splice site; AD – alternative donor / alternative 5' splice site; CE – cassette exon / exon skipping; EI – exitron; RI - retained intron. The loading control used was *UBQ1*.

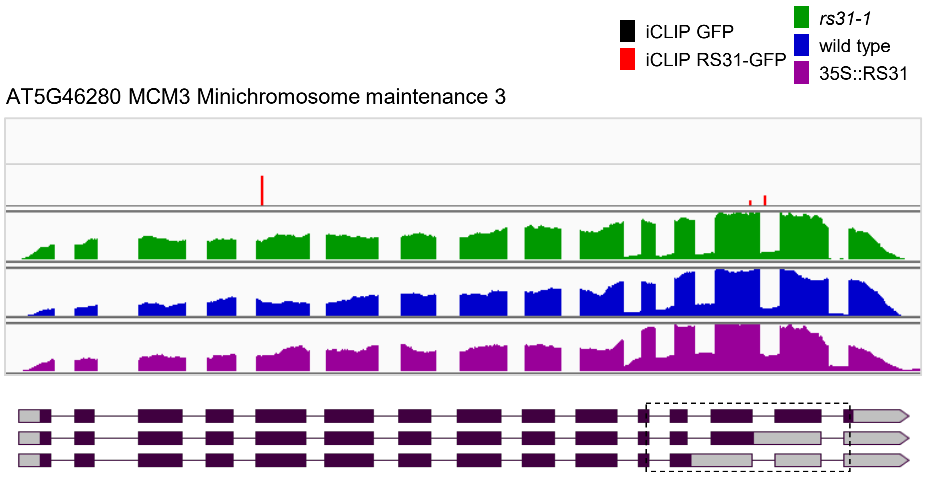

Fig. S12 Example of At-RS31 and Target of Rapamycin (TOR) pathway shared target MCM3 MINICHROMOSOME MAINTENANCE 3 (AT5G46280)

Integrated genome viewer tracks are shown for RNA sequencing read coverage in *rs31-1*, 35S::RS31 and *Arabidopsis thaliana* wild-type plants and for individual-nucleotide resolution crosslinking and immunoprecipitation (iCLIP) crosslinks for RS31-GFP and GFP control. Transcript models are visualized using Boxify tool https://boxify.boku.ac.at/. Regions from translational start codon to the reference stop codon or a premature termination codon are shown in black. Dashed rectangle denotes region undergoing differential alternative splicing in 35S::RS31 or *rs31-1* in comparison to wild-type plants. GFP – green fluorescent protein.
